# Controlling Catenation in Germanium(I) Chemistry through Hemilability

**DOI:** 10.1002/anie.202104643

**Published:** 2021-06-09

**Authors:** Alexa Caise, Liam P. Griffin, Andreas Heilmann, Caitilín McManus, Jesús Campos, Simon Aldridge

**Affiliations:** ^1^ Inorganic Chemistry Laboratory Department of Chemistry University of Oxford South Parks Road Oxford OX1 3QR UK

**Keywords:** catenation, germanium, hemilabile, low-valent compounds, pincer ligand

## Abstract

We present a novel approach for constructing chains of Group 14 metal atoms linked by unsupported metal–metal bonds that exploits hemilabile ligands to generate unsaturated metal sites. The formation/nature of catenated species (oligo‐dimetallynes) can be controlled by the use of (acidic/basic) “protecting groups” and through variation of the ligand scaffold. Reduction of Ar^NiPr2^GeCl (Ar^NiPr2^=2,6‐(^*i*^Pr_2_NCH_2_)_2_C_6_H_3_)—featuring hemilabile N^*i*^Pr_2_ donors—yields (Ar^NiPr2^Ge)_4_ (**2**), which contains a tetrameric Ge_4_ chain. **2** represents a novel type of a linear homo‐catenated Ge^I^ compound featuring unsupported E−E bonds. Trapping experiments reveal that a key structural component is the central two‐way Ge=Ge donor‐acceptor bond: reactions with IMe_4_ and W(CO)_5_(NMe_3_) give the base‐ or acid‐stabilized digermynes (Ar^NiPr2^Ge(IMe_4_))_2_ (**4**) and (Ar^NiPr2^Ge{W(CO)_5_})_2_ (**5**), respectively. The use of smaller *N*‐donors leads to stronger Ge‐N interactions and quenching of catenation behaviour: reduction of Ar^NEt2^GeCl gives the digermyne (Ar^NEt2^Ge)_2_, while the unsymmetrical system Ar^NEt2^GeGeAr^NiPr2^ dimerizes to give tetranuclear (Ar^NEt2^GeGeAr^NiPr2^)_2_ through aggregation at the N^*i*^Pr_2_‐ligated Ge^I^ centres.

## Graphical abstract

A new approach for the rational construction of chains of Group 14 metal atoms linked by unsupported metal–metal bonds is reported, which exploits hemilabile ancillary ligands to generate unsaturated metal sites. The resulting oligo‐dimetallyne chains feature alternating single and double metal–metal bonds.

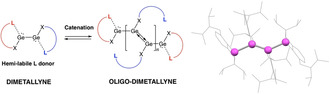

## Introduction

**Figure 1:**
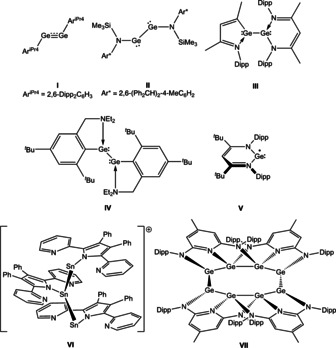
Examples of Group 14 compounds containing E−E bonds relevant to the current study [Dipp=2,6‐^*i*^Pr_2_C_6_H_3_].

Since the first example of a dimetallyne was reported some 20 years ago, the synthesis of heavier group 14 analogues of alkynes, REER (E=Si–Pb), has become a topical area within the field of molecular main group chemistry.[^1^, ^2^, ^3^] In part, this is because the isolation of dimetallynes and related systems challenged the previously held idea that multiple bonding between elements with a principle quantum number, *n*>2 was not possible.^[4]^ Moreover, due to their ambiphilic nature and the matching morphologies of their frontier orbitals, dimetallynes can interact synergistically with a number of small molecule substrates in a manner comparable to transition metal complexes.[^3^, ^5^] The first example of the activation of H_2_ by a well‐defined molecular main group compound was reported in 2005, employing a terphenyl‐stabilised digermyne, (ArGe)_2_ (**I**; Figure 1).^[6a]^ Following this seminal discovery, a number of dimetallynes have been shown to activate H_2_,^[6]^ yielding different isomeric products depending on the Group 14 element and supporting ligand system.^[6b]^ Moreover, related examples involving the activation of CO_2_,[^6g^, ^7^] chalcogens,^[8]^ P_4_,^[9]^ and organic functionalities such as alkynes^[10]^ and alkenes[^6g^, ^11^] have been reported in the past 15 years (in some cases reversibly).^[12]^

Bulky monodentate ligands offering strong σ‐donor capabilities (such as aryl or silyl donors) have been widely employed to stabilize dimetallynes kinetically with respect to aggregation processes.[^1^, ^2^, ^10b^, ^13^] Such species (e.g. **I**) typically possess short E‐E distances, but unlike alkynes, have “trans‐bent” structures and a bond order of ca. 2.[^3^, ^14^] Dimetallynes can also be stabilised thermodynamically by using bulky π‐donor substituents (such as amide,[^6c^, ^6d^, ^6e^, ^6g^, ^15^] amidinate,^[16]^ guanidinate,^[17]^ or β‐diketiminate^[18]^ ligands) or ligands that bear pendant amine^[19]^ or imine[^9^, ^19b^, ^20^] substituents (e.g. **II**–**IV**). The donation of a lone pair of electrons to each Group 14 element centre (either intramolecularly by a pincer ligand or in intermolecular fashion by an external Lewis base)^[21]^ results in population of the antibonding π* orbital and lowering of the bond order, leading to the formation of singly‐bonded species with E‐centred lone pairs.

While dimetallynes represent a key contribution to the chemistry of group 14 compounds in the formal +1 oxidation state, other isolable systems include radicals (e.g. **V**)^[22]^ and zwitterions.^[23]^ Polyhedral clusters also constitute an important contribution to low‐valent germanium chemistry,^[24]^ as do catenated chain compounds composed of E−E bonds. Metal‐metal bonded systems of the latter type are of particular interest due to potential applications in functional materials, and a diverse range of cyclic and linear heavier oligo‐alkenes has been constructed through the aggregation of divalent “ER_2_” monomers.[^6g^, ^25^] By comparison, examples of catenated systems of the type (RGe)_*n*_ featuring germanium in the +1 oxidation state are very rare.^[26]^ While a number of landmark cyclic/polycyclic systems of this composition have been reported,^[26]^ linear chain homo‐catenated systems akin to poly‐ or oligo‐acetylenes featuring alternating double and single bonds (i.e. oligo‐dimetallynes) are unprecedented. Recently, a series of linear, mixed‐valent homo‐catenated tri‐tin complexes (L^Ph^Sn)_3_X (**VI**, L^Ph^=2,5‐di(*o*‐pyridyl)‐3,4‐diphenylpyrrolate; X=Cl, AlCl_4_, OTf, PF_6_) which feature unsupported Sn‐Sn‐Sn linkages were reported by Liu et al.^[27]^ Within the realm of Ge^I^ chemistry, 2,6‐diamidopyridyl ligands have been employed to isolate a catenated octagermylene(I) complex (**VII**).^[28]^ However, this cyclic system features Ge−Ge bonds which are supported by a bridging ligand scaffold, and, to the best of our knowledge, examples of linear metal‐metal bonded chains of Ge^I^ monomers are not known (for >2 metal atoms).

**Scheme 1:**
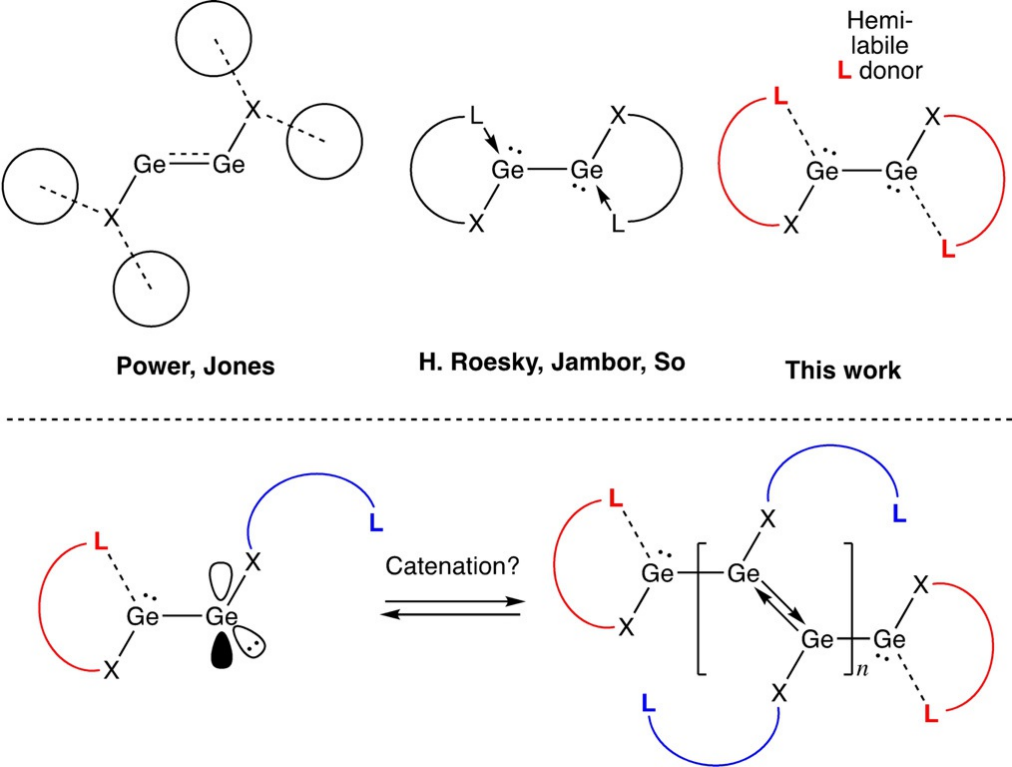
Exploiting hemilability to construct chains of unsupported E–E bonds in digermyne systems.

We hypothesized that it might be possible to construct oligo‐dimetallyne chains by employing ligands that feature pendant hemi‐labile donors which could dissociate to allow access to a reactive, low‐coordinate metal centre, and thereby bring about growth at one end of the chain (Scheme 1). With this in mind, we set out to investigate the reduction of halogermylenes ligated by 2,6‐(R_2_NCH_2_)_2_C_6_H_3_ ligands (Ar^NR2^) of differing steric bulk (R=Et, ^*i*^Pr),^[29]^ since the coordination of Lewis basic donors to low oxidation state germanium Lewis acids is known to be influenced significantly by steric factors.^[21]^

## Results and Discussion

**Figure 2:**
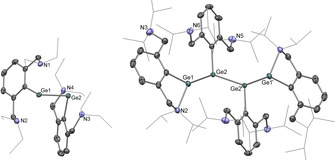
Molecular structures of (Ar^NEt2^Ge)_2_ (**1**; left) and (Ar^NiPr2^Ge)_4_ (**2**; right) as determined by X‐ray crystallography.^[42]^ Thermal ellipsoids set at the 40 % probability level. Hydrogen atoms omitted and Et/^*i*^Pr substituents shown in wireframe format for clarity. Selected interatomic distances [Å] and angles [°]: (for **1**) Ge1–Ge2 2.6066(5), Ge1⋅⋅⋅N1 2.358(2), Ge1⋅⋅⋅N2 2.695(2), Ge2⋅⋅⋅N3 2.337(2), Ge2⋅⋅⋅N4 2.794(2); (for **2**) Ge1–Ge2 2.5052(3), Ge2–Ge2′ 2.3480(4), Ge1–N2 2.2084(16), Ge1⋅⋅⋅N3 4.632(2), Ge2⋅⋅⋅N5 4.744(2), Ge2⋅⋅⋅N6 4.525(2); Ge2′‐Ge2‐Ge1 126.763(14).

**Scheme 2:**
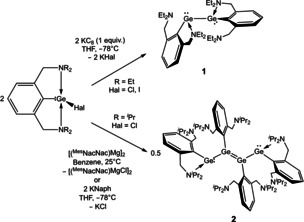
Syntheses of novel digermyne (Ar^NEt2^Ge)_2_ (**1**, top) and tetramer (Ar^NiPr2^Ge)_4_ (**2**, bottom).

Reduction of the halogermylenes Ar^NEt2^GeHal (where Hal=Cl or (better) I)^[29]^ with stoichiometric potassium graphite (KC_8_) in THF at −78 °C leads to the formation of the base‐stabilised digermyne, (Ar^NEt2^Ge)_2_ (**1**; Scheme 2). **1** can be isolated as orange crystals in 73 % yield and has been characterised by standard spectroscopic and analytical techniques, and its molecular structure determined by X‐ray crystallography (Figure 2). The Ge‐Ge bond length (2.6066(5) Å) is characteristic of Ge‐Ge single bonds, and lies within the range reported for related species (LGeGeL (**II**): 2.7093(7) Å; LGeGeL′ (**III**): 2.5498(8) Å).[^6c^, ^18a^] As reported for the related distannyne, (Ar^NMe2^Sn)_2_,^[19a]^ each metal centre is coordinated by both intramolecular *N*‐donors (albeit to an unsymmetrical extent); the Ge−N bond lengths vary from 2.337(2) Å (for Ge2‐N3) to 2.794(2) Å (for Ge2‐N4) and are of comparable length to those measured for the chlorogermylene precursor (e.g. Ar^NEt2^GeCl: 2.337(11), 2.570(11) Å).^[29]^ The ^1^H NMR spectrum of **1** features a single set of ligand signals, implying equivalence on the NMR timescale, and suggesting that it retains its formulation as a digermyne in solution. As such, the behaviour of the NEt_2_‐functionalized system is in line with previous reports of isolated Ge‐Ge bond formation under reductive conditions.

By contrast, reduction of the bulkier Ar^NiPr2^GeCl precursor with stoichiometric [(^Mes^NacNac)Mg]_2_ (^Mes^Nacnac=(MesNCMe)_2_CH) in benzene, or potassium naphthalenide (KNaph) in tetrahydrofuran, leads to more extensive E‐E bond formation. The product isolated after recrystallization from benzene at room temperature is not the corresponding digermyne, (Ar^NiPr2^Ge)_2_. Instead, analysis by single‐crystal X‐ray diffraction reveals that it is a novel centro‐symmetric tetramer, consisting of four germanium(I) centres linked by unsupported metal‐metal bonds, that is, (Ar^NiPr2^Ge)_4_ (**2**) (Scheme 2 and Figure 2).^[30]^ The Ge_4_ chain consists of alternating single and double Ge‐Ge bonds. The Ge1‐Ge2/Ge1′‐Ge2′ bond length (2.5052(3) Å) is characteristic of a single bond,[^18a^, ^19a^] while the Ge2‐Ge2′ separation (2.3480(4) Å) and associated *trans*‐bent geometry is indicative of a two‐way donor‐acceptor interaction (analogous to that found—archetypally—in Lappert's digermene and distannene),[^31^, ^32^, ^33^] arising from donation of the lone pair of one germanium centre into the vacant *p*‐orbital of the other (and vice versa). The four germanium centres lie in the same plane (torsion angle, 180°) and the Ge‐N distances fall into two distinct categories. While the terminal germanium atoms (i.e. Ge1/Ge1′) feature coordination of one of the amino side‐arm donors (*d*(Ge1‐N2)=2.2084(16) Å), the internal metal centres (i.e. Ge2) are not coordinated by either *N*‐donor (Ge‐N contacts of >4.5 Å), as the relevant germanium *p*‐orbitals are used instead for the construction of the central Ge=Ge linkage.

The structural differences between (dimeric) **1** and (tetrameric) **2** are thought to reflect differing *N*‐donor coordination capabilities, with the N→Ge interaction in the latter case being of comparable (or lower) thermodynamic value compared to Ge=Ge bond formation (see below). In theory, this weak *N*‐donor behaviour might offer the possibility for extending the chain further at the terminal germanium centres of **2** if the N→Ge bond associated with Ge1/Ge1′ were itself labile. Interestingly though, this bond is very short (2.2084(16) Å), compared (for example) to comparable contacts in **1** (2.358(2) and 2.337(2) Å), implying that it effectively acts as a “cap” on further chain growth in this system. We hypothesize that this strengthening of N→Ge coordination at Ge1 is associated with electron‐withdrawal from the Ge1‐Ge2 moiety caused by Ge2 behaving as a Lewis base (electron donor). In the case of **2**, this electron donation (to Ge2′) helps establish the central two‐way donor acceptor bond between Ge2 and Ge2′, but we could also establish this as a more general phenomenon by coordination to an iron‐centred Lewis acid.

**Scheme 3:**
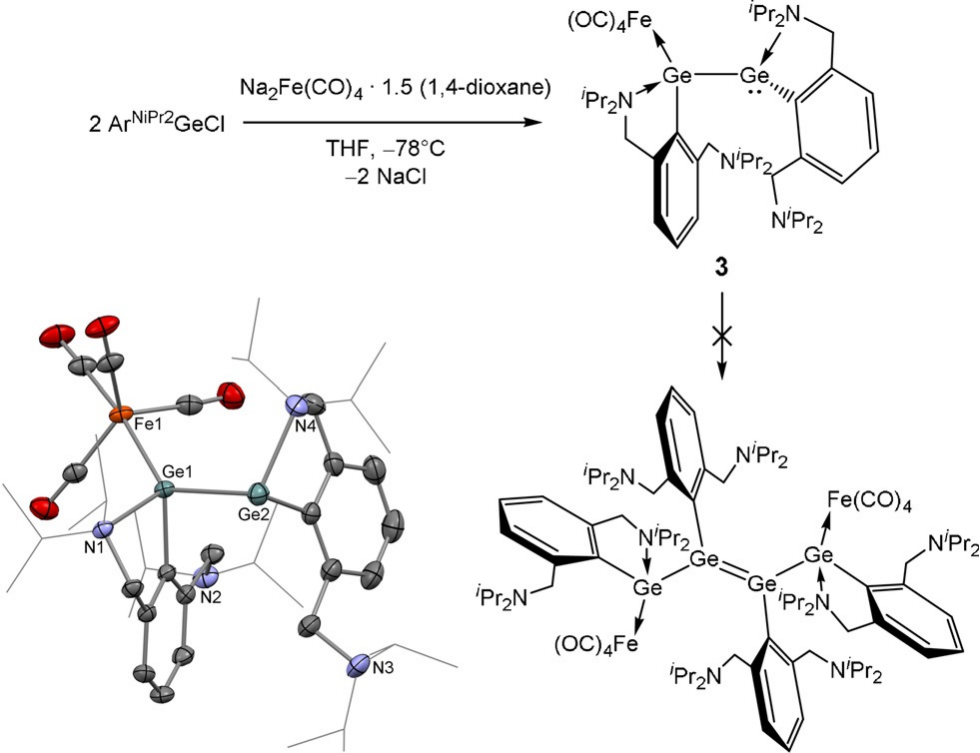
Synthesis of unsymmetrically coordinated germanium(I)‐iron complex (Ar^NiPr2^Ge)_2_Fe(CO)_4_ (**3**) and the molecular structure of **3** as determined by X‐ray crystallography (bottom left).^[42]^ Thermal ellipsoids set at the 40 % probability level. Hydrogen atoms and *n*‐hexane solvate molecule omitted and ^*i*^Pr substituents shown in wireframe format for clarity. Selected interatomic distances [Å] and angles [°]: Ge1–Ge2 2.5974(5), Ge1–Fe1 2.4301(5), Ge1–N1 2.259(2), Ge1⋅⋅⋅N2 4.840(3), Ge2⋅⋅⋅N3 4.571(4), Ge2–N4 2.191(3); Fe1‐Ge1‐Ge2 134.415(18).

Accordingly, following an approach developed by Mak and co‐workers,^[15]^ we prepared the unsymmetrically‐coordinated germanium(I)‐iron complex (Ar^NiPr2^Ge)_2_Fe(CO)_4_ (**3**), via the reduction of Ar^NiPr2^GeCl with Collman's reagent, Na_2_Fe(CO)_4_, in THF. **3** can be isolated as orange crystals, albeit in relatively low (14 %) isolated yield. The molecular structure (Scheme 3) shows that the [Fe(CO)_4_] fragment is coordinated to a single germanium centre (Ge1), while Ge2 is three‐coordinate and bears an uncomplexed lone pair. The Ge1‐Fe distance (2.4301(5) Å) is marginally longer than those of previously reported examples (2.340(1)–2.400(1) Å), while the Ge1‐Ge2 separation (2.5974(5) Å) is consistent with the presence of a single Ge−Ge bond.[^15^, ^34^] Of most relevance is the very close approach of one of the pendant *N*‐donors to Ge2 (2.191(3) Å), in line with the idea that coordination of the remote germanium centre (Ge1) to the [Fe(CO)_4_] Lewis acid enhances the electrophilicity of Ge2. Consistently, **3** shows no propensity for oligomerization via Ge2 in either solid or solution phases, suggesting that (as with the terminal germanium centres in **2**) the more tightly bound amine donor acts as a “cap” on chain growth.

From a broader perspective, the formation of a chain of four Ge^I^ atoms in **2** is highly unusual and represents a very rare example of linear catenation among the heavier Group 14 compounds—particularly because it involves metal‐metal bonds which are not additionally supported by a bridging ligand.[^26^, ^27^, ^28^, ^35^] Simple catenation is typically disfavoured due to entropic factors and the increasingly weak nature of the E‐E bonds upon descending the group. With this in mind, we were keen to explore how aggregation of this type might be controlled. Conceivably, **2** arises through transient formation of a base‐stabilised digermyne analogous to **1** (i.e. (Ar^NiPr2^Ge)_2_), with subsequent tetramer formation being contingent on access (thermally) to both a lone pair and a vacant orbital at one of the germanium termini. We sought to investigate this hypothesis further by probing the behaviour of the system in the presence of external (Lewis) acidic and basic traps.

**Figure 3:**
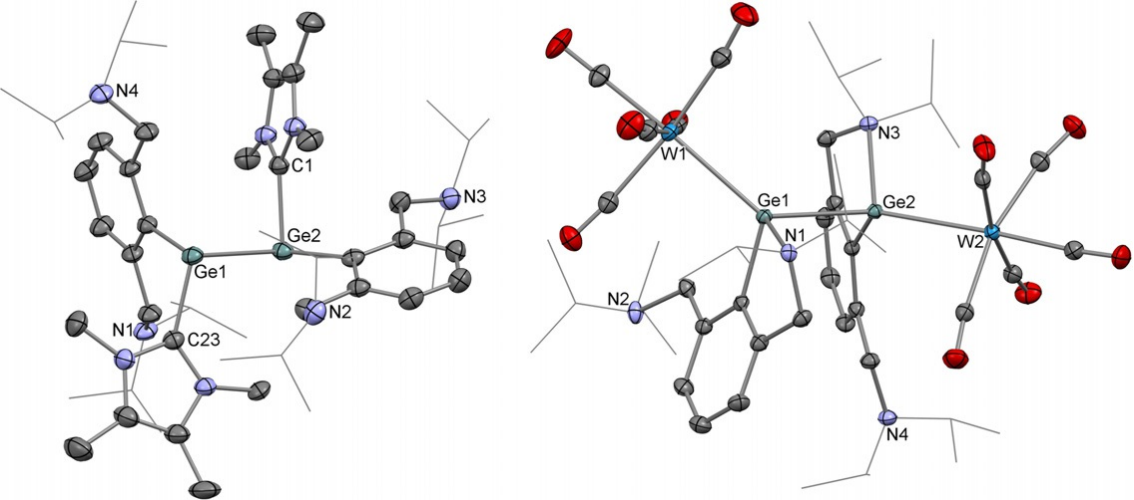
Molecular structures of (Ar^NiPr2^Ge(IMe_4_))_2_ (**4**; left) and (Ar^NiPr2^Ge{W(CO)_5_})_2_ (**5**; right) as determined by X‐ray crystallography.^[42]^ Thermal ellipsoids set at the 40 % probability level. Hydrogen atoms and (for **5**) benzene solvate molecules omitted and ^*i*^Pr substituents shown in wireframe format for clarity. Selected interatomic distances [Å] and angles [°]: (for **4**) Ge1–Ge2 2.5378(6), Ge1–C23 2.066(3), Ge2–C1 2.046(3), Ge1⋅⋅⋅N1 4.901(3), Ge1⋅⋅⋅N4 4.452(3), Ge2⋅⋅⋅N2 4.863(4), Ge2⋅⋅⋅N3 4.468(4); Ge1‐Ge2‐C1 91.01(9), C23‐Ge1‐Ge2 101.80(10); (for **5**) Ge1–Ge2 2.7287(3), Ge1–W1 2.7741(3), Ge2–W2 2.7678(3), Ge1–N1 2.2054(17), Ge1⋅⋅⋅N2 4.880(2), Ge2–N3 2.2042(17), Ge2⋅⋅⋅N4 4.895(2); Ge2‐Ge1‐W1 131.057(10), Ge1‐Ge2‐W1 130.964(10).

**Scheme 4:**
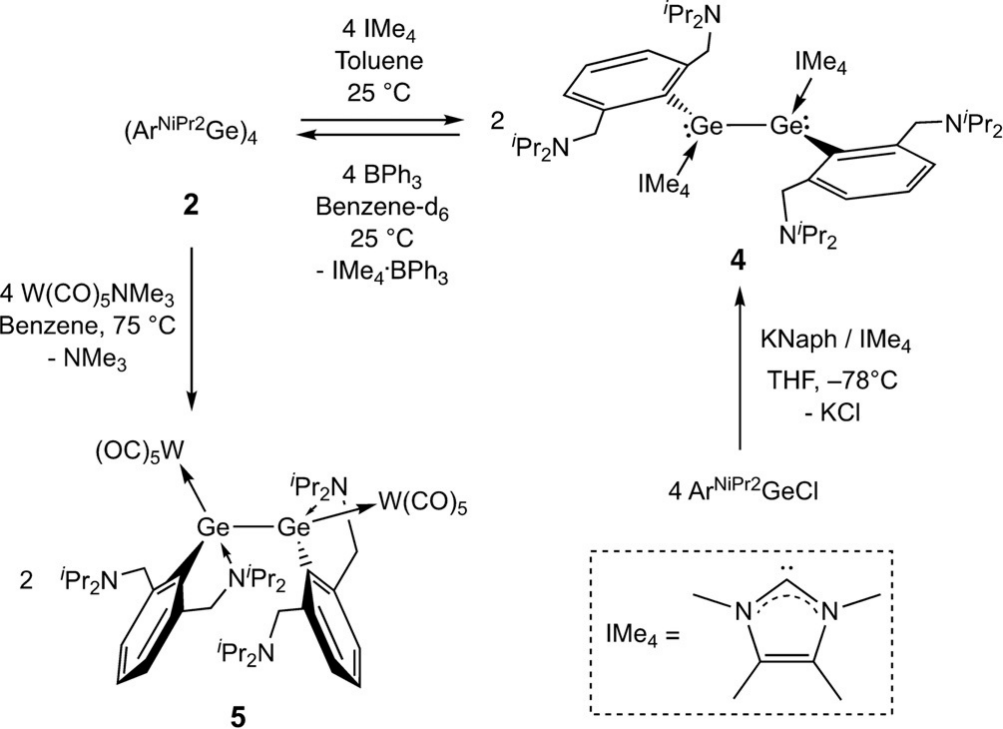
Syntheses of doubly base‐ and acid‐stabilized digermynes (Ar^NiPr2^Ge(IMe_4_))_2_ (**4**) and (Ar^NiPr2^Ge{W(CO)_5_})_2_ (**5**). In the case of **4**, removal of the carbene ligands by reaction with BPh_3_ regenerates tetrameric **2**.

The reaction of the Ge_4_ tetramer **2** with four equivalents of the N‐heterocyclic carbene (NHC) 1,3,4,5‐tetramethylimidazol‐2‐ylidene (IMe_4_) in toluene proceeds at room temperature to yield the base‐stabilised digermyne (Ar^NiPr2^Ge(IMe_4_))_2_ (**4**: Scheme 4). **4** can also be prepared by the reduction of an equimolar mixture of Ar^NiPr2^GeCl and IMe_4_ in THF with stoichiometric potassium naphthalenide; analysis of the reaction mixture by ^1^H NMR spectroscopy shows that the reaction proceeds selectively, with this being the only Ar^NiPr2^‐containing product seen in solution. **4** has been characterised by standard analytical and spectroscopic techniques, in addition to X‐ray crystallography (Figure 3). Its molecular structure confirms the connectivity suggested by spectroscopic measurements, and features a Ge−Ge bond length (2.5378(6) Å) which lies between that of **1** and the terminal Ge−Ge bond of tetrameric **2**. As expected, coordination of the stronger carbene donors to the germanium centres of **4** induces dissociation of the hemi‐labile amino donors, with all Ge‐N contacts being >4.4 Å in length.

Removal of the carbene donors from **4** by treatment with a borane Lewis acid regenerates tetrameric **2**, thereby establishing the reversibility of dimer/tetramer inter‐conversion. Addition of two equivalents of triphenylborane to a solution of **4** in [D_6_]benzene at room temperature leads to an immediate colour change from orange‐red to dark red. The ^1^H and ^11^B NMR spectra of the reaction mixture show that **4** is quantitatively consumed (within 5 min), with concomitant formation of the Lewis adduct, IMe_4_⋅BPh_3_.^[36]^ Large crystals of the germanium‐containing product could be grown from the reaction solution, the identity of which was confirmed by X‐ray crystallography as tetrameric **2**.

The behaviour of **2** in the presence of Lewis acids was also examined: heating a mixture of (Ar^NiPr2^Ge)_4_ and W(CO)_5_(NMe_3_) (4 equiv) in [D_6_]benzene to 75 °C for 20 h leads to cleavage of the tetrameric chain and coordination of a [W(CO)_5_] fragment to each germanium centre of the (Ar^NiPr2^Ge)_2_ dimer.[^37^, ^38^] (Ar^NiPr2^Ge{W(CO)_5_})_2_ (**5**) has been characterized by standard spectroscopic/analytical techniques and by X‐ray crystallography (Figure 3). Each germanium centre is coordinated to one [W(CO)_5_] fragment and by one pendant *N*‐donor; the Ge‐N distances (*d*(Ge1‐N1)=2.2054(17); *d*(Ge2‐N3)=2.2042(17) Å) are comparable to that measured for **2** (2.2084(16) Å). On the other hand, the Ge‐W distances (2.7741(3) and 2.7678(3) Å) are significantly longer than those reported for examples featuring related ligands (e.g. Ar^NEt2^Ge(OH)W(CO)_5_ *d*(Ge‐W) 2.595(0) Å; ArGe(Cl)W(CO)_5_ (Ar=2,4‐di‐tert‐butyl‐6‐(diisopropylaminomethyl)phenyl) *d*(Ge‐W) 2.658(1) Å),[^39^, ^40^] presumably reflecting (at least in part) increased steric loading at germanium. Consistently, the Ge−Ge bond length (2.7287(3) Å) is very much longer than that of carbene complex **4** (2.5378(6) Å) and the terminal Ge−Ge bond length of **2** (2.5052(3) Å), and lies at the upper limit of Ge−Ge bond lengths reported for digermynes.^[6c]^ Coordination of Lewis bases to digermynes and the (typical) subsequent reduction in their bond order has been well studied.[^11a^, ^21^] By contrast, the impact of the coordination of digermynes to Lewis acids is less well established and, prior to this work, crystallographically characterized examples of such complexes were limited to two germanium(I)‐iron complexes: [L{Fe(CO)_4_}GeGeL] (L=N(SiMe_3_)C(Ph)C(SiMe_3_)(C_5_H_4_N‐2))^[15]^ and [LGe{Fe(CO)_4_}Ge{Fe(CO)_4_}L] (L=PhC(N^t^Bu)_2_).^[34]^ Interestingly, coordination of the digermyne to the Fe(CO)_4_ fragment(s) leads to no significant change in the Ge−Ge bond length in both cases.

Trapping of the (Ar^NiPr2^Ge)_2_ dimer by both Lewis acids and Lewis bases (and re‐aggregation when the trap is removed) is consistent with the idea that aggregation to give tetrameric **2** occurs via a two‐way donor/acceptor motif, requiring the presence of both a lone pair and vacant orbital at germanium. Moreover, the differing propensities for tetramer formation as a function the *N*‐donor substituent ((Ar^NEt2^Ge)_2_ vs. (Ar^NiPr2^Ge)_4_) suggest that the strength of binding of the pendant *N*‐donors influences whether catenation proceeds. We hypothesize that, for the N^*i*^Pr_2_ system, the increased size of the R substituents renders the *N*‐donor less strongly donating to the (sterically encumbered) germanium centre.^[21]^ The measured Ge‐N distances for the precursor chlorogermylenes, Ar^NR2^GeCl (R=Et, ^*i*^Pr), provide some support for this hypothesis. As such, while one of the Ge‐N distances is similar in both systems, the other is much longer for R=^*i*^Pr (*d*(Ge‐N) 2.315(2), 2.892(2) Å; R=Et: *d*(Ge‐N) 2.337(11), 2.570(10) Å), suggesting that the Ge‐N interaction becomes weaker in the N^*i*^Pr_2_ system as the metal centre becomes more crowded.^[28]^ On this basis, we postulate that R=Et/^*i*^Pr represents a crossover point between “typical” and catenation behaviour for the Ge^I^ systems.

**Scheme 5:**
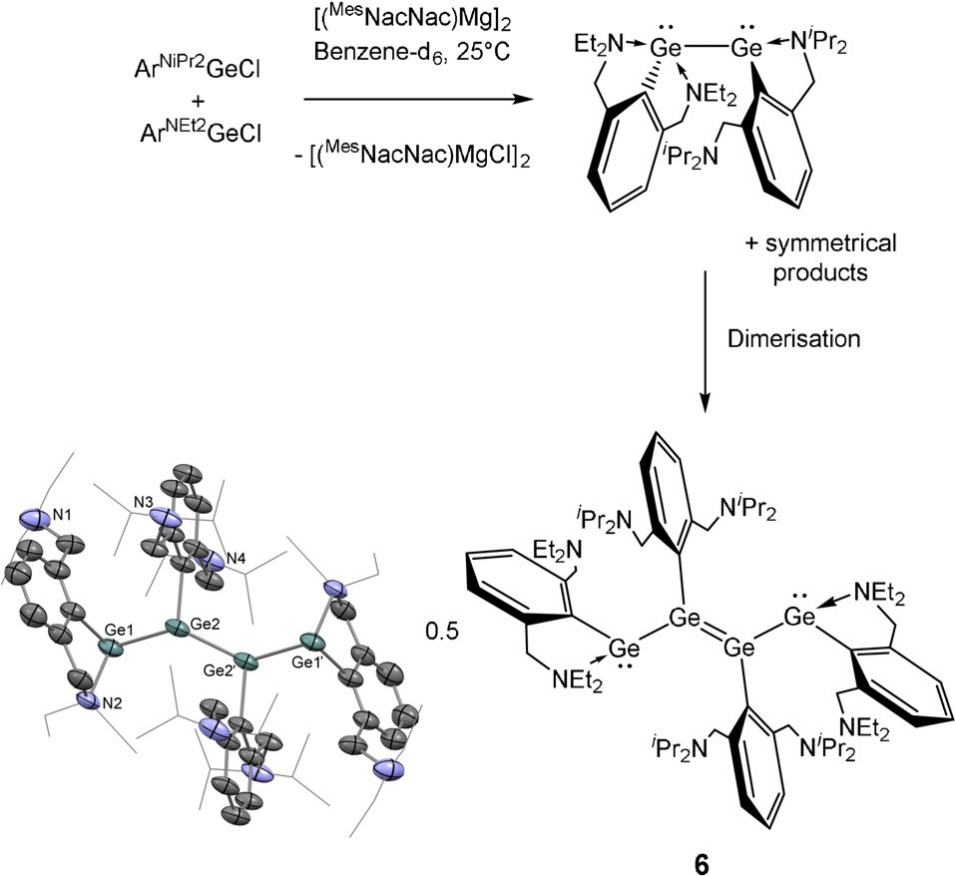
Synthesis and molecular structure of (Ar^NEt2^GeGeAr^NiPr2^)_2_ (**6**) as determined by X‐ray crystallography.^[42]^ Thermal ellipsoids set at the 40 % probability level. Hydrogen atoms omitted and ^*i*^Pr substituents shown in wireframe format for clarity. Selected interatomic distances [Å] and angles [°]: Ge1–Ge2 2.5068(8), Ge2–Ge2′ 2.3204(12), Ge1⋅⋅⋅N1 4.597(5), Ge1–N2 2.149(4), Ge2⋅⋅⋅N3 4.513(6), Ge2⋅⋅⋅N4 4.717(6); Ge2′‐Ge2‐Ge1 129.87(3).

This hypothesis suggests that the unsymmetrical digermyne Ar^NEt2^GeGeAr^NiPr2^—if accessible—might selectively direct chain growth to occur at the isopropyl‐ligated germanium centre to form the Ar^NEt2^‐capped tetranuclear system Ar^NEt2^GeGe(Ar^NiPr2^)Ge(Ar^NiPr2^)GeAr^NEt2^. Indeed, this can be achieved in practice by the reduction of an equimolar mixture of Ar^NEt2^GeCl and Ar^NiPr2^GeCl with stoichiometric [(^Mes^NacNac)Mg]_2_ in [D_6_]benzene. While the reaction is not selective (the formation of (Ar^NEt2^Ge)_2_ is observed by ^1^H NMR spectroscopy),^[41]^ analysis by X‐ray crystallography of the dark red crystals carefully grown from the reaction solution confirm the formation of (Ar^NEt2^GeGeAr^NiPr2^)_2_ (**6**; Scheme 5), in which both of the central Ge atoms are ligated by the Ar^NiPr2^ ligand. While **2** and **6** are essentially isostructural, the Ge2‐Ge2′ and Ge1‐N2 distances of **6** (2.3204(12), 2.149(4) Å, respectively) are marginally shorter than those of **2** (2.3480(4), 2.2084(16) Å, respectively). The formation of **6**—while not synthetically selective—presents strong evidence that catenation behaviour can be directed to a particular site based on the steric bulk of the amino substituents.

## Conclusion

Reduction of the chlorogermylene, Ar^NiPr2^GeCl, featuring an aryl ligand with pendant hemi‐labile N^*i*^Pr_2_ donors leads to the formation of (Ar^NiPr2^Ge)_4_ (**2**) featuring a tetrameric chain of Ge^I^ units. **2** represents an extremely rare example of a homo‐catenated compound featuring unsupported Ge‐Ge bonds containing germanium in the formal oxidation state +1. The formation of **2**, via a two‐way donor/acceptor interaction between the two central germanium atoms, is dependent on access to both a Ge‐centred lone pair and a vacant orbital in the putative digermyne, (Ar^NiPr2^Ge)_2_, as demonstrated by trapping experiments with Lewis acids/bases. Reactions with IMe_4_ and W(CO)_5_(NMe_3_) give the doubly base‐ or acid‐stabilized digermynes (Ar^NiPr2^Ge(IMe_4_))_2_ (**4**) and (Ar^NiPr2^Ge{W(CO)_5_})_2_ (**5**), respectively. In the case of **4**, removal of the carbene ligands by reaction with BPh_3_ leads to the regeneration of tetrameric **2**. Constraining the steric profile of the ancillary *N*‐donors leads to stronger Ge‐N interactions and to quenching of catenation behaviour. Reduction of Ar^NEt2^GeCl leads to the formation of the simple digermyne (Ar^NEt2^Ge)_2_, while the unsymmetrical digermyne, Ar^NEt2^GeGeAr^NiPr2^, dimerizes to give tetranuclear (Ar^NEt2^GeGeAr^NiPr2^)_2_, with aggregation occurring at the isopropyl‐ligated germanium centres.

As such, we have demonstrated a novel approach for generating chains of metal atoms featuring unsupported metal‐metal bonds, by making use of hemi‐labile ancillary ligands to promote catenation. We have also demonstrated that we can exercise control over the formation/nature of such species by the use of (acidic or basic) protecting groups and through modification of the supporting ligands. Extension of this approach to generate longer metal chains through further ligand modification is currently being investigated.

## Conflict of interest

The authors declare no conflict of interest.

## Supporting information

As a service to our authors and readers, this journal provides supporting information supplied by the authors. Such materials are peer reviewed and may be re‐organized for online delivery, but are not copy‐edited or typeset. Technical support issues arising from supporting information (other than missing files) should be addressed to the authors.

Supplementary
