# Supplementary material for: Controlling Catenation in Germanium(I) Chemistry through Hemilability
Source: Angew Chem Int Ed Engl. 2021 Jun 9;60(28):15606–12. doi: 10.1002/anie.202104643 (PMC8362110; doi:10.1002/anie.202104643)
Supplement: Supplementary file 1 — Supplementary [file ANIE-60-15606-s001.pdf]

Angewandte Chemie
Eine Zeitschrift der Gesellschaft Deutscher Chemiker

Supporting Information

Controlling Catenation in Germanium(I) Chemistry through Hemilability

*Alexa Caise, Liam P. Griffin, Andreas Heilmann, Caitilín McManus, Jesús Campos, and Simon Aldridge**

anie_202104643_sm_miscellaneous_information.pdf

Supporting Information

Table of Contents (19 pages)

(i)	General considerations	s2
(ii)	Starting materials	s2
(iii)	Novel compounds	s3
(iv)	^{1}H and ^{13}C NMR spectra of novel compounds	s8
(v)	X-ray crystallographic studies	s15
(vi)	References	s19

(i) General considerations

All manipulations were carried out using standard Schlenk line or dry-box techniques under an atmosphere of dry argon or dinitrogen. Solvents were degassed by sparging with argon and dried by passing through a column of the appropriate drying agent using a commercially available Braun SPS and stored over potassium. Diethyl ether and THF were dried over and distilled from sodium and benzophenone/potassium (respectively) and stored over a potassium mirror or sodium mirror, respectively. THF employed in the preparation of $(Ar^{NEt2}Ge)_2$ was dried over and distilled from potassium and degassed by three freeze-pump-thaw cycles; in this case the solvent was *not* stored over a sodium mirror. Hexamethyldisiloxane was dried over CaH_2, distilled, degassed by three freeze-pump-thaw cycles and stored over activated 4 Å molecular sieves. NMR spectra were measured in benzene-d_6 which was dried over CaH_2, with the solvent then being distilled under reduced pressure, degassed by three freeze-pump-thaw cycles and stored under argon in Teflon valve ampoules. NMR samples were prepared under argon in 5 mm Wilmad 507-PP tubes fitted with J. Young Teflon valves. NMR spectra were measured on a Bruker Avance III HD Nanobay 400 MHz NMR spectrometer equipped with a 9.4 T magnet, Bruker Avance III 500 MHz NMR spectrometer equipped with a 11.75 T magnet or a Bruker Avance III NMR 500 MHz NMR spectrometer equipped with a 11.75 T magnet and a ^{13}C detect cryoprobe. ^{1}H and ^{13}C NMR spectra were referenced internally to residual protio-solvent (^{1}H) or solvent (^{13}C) resonances and are reported relative to tetramethylsilane (δ = 0 ppm). Chemical shifts are quoted in δ (ppm) and coupling constants in Hz. Infra-red spectra were measured on a Nicolet 500 FT-IR spectrometer. Samples were measured as a Nujol mull and were prepared inside a glovebox before being sealed in an airtight cell. Elemental analyses were carried out at London Metropolitan University or by Elemental Microanalysis Ltd., Okehampton, Devon.

(ii) Starting materials

The syntheses of 2,6-$(BrCH_2)_2C_6H_3Br$,[S1] [2,6-$(R_2NCH_2)_2C_6H_3$]GeCl (R = Et, ^{i}Pr),[S2] $(^{Mes}NacNacMg)_2$,[S3] potassium naphthalenide (KNaph),[S4] KC_8,[S5] $W(CO)_5NMe_3$,[S6] 1,3,4,5-tetramethylimidazol-2-ylidene[S7] (IMe_4) were carried out as per literature precedent. All other reagents were used as received.

Synthesis of [2,6-($^{i}Pr_2NCH_2)_2C_6H_3$]GeCl (Ar^{NiPr2}GeCl): The preparation of Ar^{NiPr2}GeCl was undertaken following the method reported by Couret *et al.*[S2] Single crystals which were suitable for X-ray diffraction could be obtained from the storage of a concentrated solution of Ar^{NiPr2}GeCl in toluene at −30 °C.

(iii) Novel compounds

Synthesis of Ar^{NiPr2}GeI: To a solution of Ar^{NiPr2}GeCl (0.5 g, 1.21 mmol) in toluene (7 mL) at room temperature was added dropwise neat trimethylsilyl iodide (Me_3SiI) (0.2 mL, 1.41 mmol). The resulting yellow solution was stirred for 2 h and volatiles were subsequently removed *in vacuo* to yield a sticky orange solid. The solid was re-extracted into minimal toluene, filtered and concentrated to the point of incipient crystallisation. Storage of this solution at −30 °C overnight yielded large colourless crystals, which were suitable for X-ray diffraction. The crystals were isolated and dried *in vacuo*. Further concentration of the solution, followed by storage at −30 °C, yielded a second crop of crystals. Combined yield: 0.49 g (80%). **Spectroscopic data: ^{1}H NMR** (400 MHz, benzene-d$_6$, 298 K): δ_H 1.01 (12H, d, $^3J_{HH}$ = 6.7 Hz, CH_3 of ^{i}Pr), 1.06 (12H, d, $^3J_{HH}$ = 6.7 Hz, CH_3 of ^{i}Pr), 3.42 (4H, sept, $^3J_{HH}$ = 6.7 Hz, CH of ^{i}Pr), 3.74 and 4.05 (4H, AB system, $^2J_{HH}$ = 14.6 Hz, CH_2N), 6.94 (2H, d, $^3J_{HH}$ = 7.5 Hz, *m*-CH of Ar), 7.11 ppm (1H, t, $^3J_{HH}$ = 7.5 Hz, *p*-CH of Ar). **^{13}C{^{1}H} NMR** (126 MHz, benzene-d$_6$, 298 K): δ_C 21.0, 21.8 (CH$_3$ of ^{i}Pr), 51.8 (CH of ^{i}Pr), 55.2 (CH$_2$N), 123.6 (*m*-Ar*C*), 128.6 (*p*-Ar*C*), 147.7 (*o*-Ar*C*), 158.3 (*ipso*-Ar*C*). **Elemental microanalysis**: calc. for $C_{20}H_{35}N_2GeI$ C 47.75%, H 7.01%, N 5.57%, meas. C 47.85%, H 7.22%, N 5.50%.

Synthesis of Ar^{NEt2}GeI: Method A: To a solution of 2,6-(Et_2NCH_2)$_2C_6H_3Br$ (3.36 g, 10.3 mmol) in diethyl ether (20 mL) at 0 °C was added dropwise ^{n}BuLi (7.7 mL, 12.32 mmol, 1.6 M in *n*-hexane). The resulting solution was allowed to warm to room temperature and stirred for 30 min during which time the solution became pale yellow in colour and precipitated crystalline 1-lithio-2,6-bis[(diethylamino)methyl])benzene. Volatiles were subsequently removed *in vacuo* and the resulting crystalline material was dissolved in tetrahydrofuran (25 mL). The solution was then added dropwise to a solution of $GeCl_2$·dioxane (2.62 g, 11.3 mmol), also in THF (20 mL), at –78 °C before being allowed to warm to room temperature and stirred for 20 min. Volatiles were then removed *in vacuo* and the resulting oily residue treated with *n*-hexane (100 mL), filtered and concentrated to *ca.* 25 mL. Neat trimethylsilyliodide (1.75 mL, 12.3 mmol) was added dropwise to the solution which, after stirring for 10 min, began to precipitate bright yellow crystalline material. The resulting slurry was stirred for 1 h at room temperature before being concentrated to *ca.* 10 mL. The crude product was isolated by filtration, dried *in vacuo*, extracted into hot hexane (120 mL, 60 °C) and filtered whilst hot. The solution was allowed to cool to room temperature and then concentrated to the point of incipient crystallisation. Storage of this solution at –30 °C overnight yielded large yellow crystals, which were suitable for X-ray crystallography. Further concentration of the supernatant solution, followed by storage at –30 °C, yielded a second crop of crystals. Combined yield: 3.92 g (85%). **Method B:** To a Schlenk tube containing Ar^{NEt2}GeCl (0.5 g, 1.41 mmol) and NaI (0.25 g, 1.67 mmol) was added THF (10 mL). The resulting slurry was stirred for 18 h at room temperature during which time the colourless solution evolved a bright yellow colour. The slurry was subsequently filtered and volatiles were removed *in vacuo* to yield a sticky yellow residue which was extracted into *n*-hexane (50 mL). The resulting slurry was filtered and concentrated to the point of incipient crystallisation. Storage of this solution at 6 °C for 2 h and then at −30 °C overnight yielded large yellow crystals of Ar^{NEt2}GeI, which were isolated and dried *in vacuo*. Yield: 0.52 g (83%). **^{1}H NMR** (400 MHz, benzene-d$_6$, 298 K): δ_H 0.83 (12H, t, $^3J_{HH}$ = 7.2

Hz, CH_3 of Et), 2.61 – 2.80 (8H, br m, overlapping CH_2 of Et), 3.39 and 3.71 (4H, AB system, $^2J_{HH}$ = 14.7 Hz, CH_2N), 6.89 (2H, d, $^3J_{HH}$ = 7.4 Hz, *m*-C*H* of Ar), 7.10 (1H, t, $^3J_{HH}$ = 7.4 Hz, *p*-C*H* of Ar). **$^{13}C\{^1H\}$ NMR** (101 MHz, benzene-d$_6$, 298 K): δ_C 9.8 (CH_3 of Et), 45.1 (CH_2 of Et), 59.1 (CH_2N), 123.4 (*m*-Ar*C*), 128.6 (*p*-ArC), 145.3 (*o*-Ar*C*), 154.7 (*ipso*-Ar*C*). **Elemental microanalysis**: calc. for $C_{16}H_{27}N_2GeI$ C 43.00%, H 6.09%, N 6.27%, meas. C 43.08%, H 6.08%, N 6.30%.

Synthesis of ($Ar^{NEt2}Ge)_2$ (1): A rapidly stirred mixture of $Ar^{NEt2}GeI$ (500 mg, 1.12 mmol) and KC_8 (174 mg, 1.29 mmol) was cooled to –78 °C and to it was added tetrahydrofuran (3.5 mL). The resulting slurry was stirred for 5 min at –78 °C before being allowed to warm to room temperature and stirred for a further 20 min. Following this, volatiles were removed *in vacuo* and the resulting residue was treated with hexamethyldisiloxane (HMDSO) (10 mL). The resulting orange solution was filtered and concentrated until orange oil was deposited onto the walls of the Schlenk tube (to *ca.* 5 mL). The oil was redissolved and the solution stored at –30 °C overnight. After allowing the solution to warm again to room temperature, small orange crystals of $(Ar^{NEt2}Ge)_2$ began to grow from the solution. After storage of the solution overnight at room temperature, the crystals were isolated and dried *in vacuo*. Further concentration of the supernatant solution, followed by storage at –30 °C overnight and then at room temperature for a further 24 h yielded a second crop of crystals. Combined yield: 263 mg (73%). Single crystals of X which were suitable for X-ray crystallography were obtained from the storage of a concentrated solution of **1** in HMDSO at 6 °C for 3 d. **^1H NMR** (400 MHz, benzene-d$_6$, 298 K): δ_H 0.93 (24H, apparent t, $^3J_{HH}$ = 7.1 Hz, overlapping CH_3 of Et), 2.71 – 2.86 (16H, m, overlapping CH_2 of Et), 3.09 and 3.62 (8H, AB system, $^2J_{HH}$ = 14.0 Hz, CH_2N), 7.10 – 7.19 (6H, m, *m*- and *p*-C*H* of Ar). **$^{13}C\{^1H\}$ NMR** (101 MHz, benzene-d$_6$, 298 K): δ_C 10.8 (CH_3 of Et), 46.8 (CH_2 of Et), 61.7 (CH_2N), 123.2 (*m*-Ar*C*), 124.3 (*p*-Ar*C*), 142.1 (*o*-Ar*C*), 163.4 (*ipso*-Ar*C*). **Elemental microanalysis**: calc. for $C_{32}H_{54}N_4Ge$ C 60.05%, H 8.50%, N 8.75%, meas. C 59.96%, H 8.66%, N 8.75%. Preparation of $(Ar^{NEt2}Ge)_2$ in an analogous manner employing the chlorogermylene, $Ar^{NEt2}GeCl$, is possible but the product is isolated in lower yield and purity.

Synthesis of $(Ar^{NiPr2}Ge)_4$ (2): Method A: To a rapidly stirred mixture of $Ar^{NiPr2}GeCl$ (600 mg, 1.46 mmol) and $(^{Mes}NacnacMg)_2$ (573 mg, 0.80 mmol) was added benzene (20 mL). The reaction mixture was stirred for 3 h at room temperature, during which time the solution evolved a deep red colour and a colourless powder was precipitated. The solution was filtered and stored at room temperature for 3 d, yielding single, dark red crystals which were suitable for X-ray crystallography. The crystals were isolated, washed with pentane (2 mL) and dried *in vacuo*. Yield: 323 mg (59%). **Method B:** To a solution of $Ar^{NiPr2}GeCl$ (300 mg, 0.73 mmol) in THF (3 mL) at –78 °C was added dropwise a solution of potassium naphthalenide (KNaph) (146 mg, 0.87 mmol), also in THF (6 mL). Upon completion of the dropwise addition, the deep red solution was allowed to warm to room temperature and was stirred for 1 h. Volatiles were subsequently removed *in vacuo* to yield a violet solid, which was treated with hot benzene (40 mL, 70 °C). The solution was filtered (while hot), and slowly allowed to cool to room temperature over the course of several hours, yielding small, dark red crystals of $(Ar^{NiPr2}Ge)_4$. These crystals were isolated and dried *in vacuo*, and further concentration of the supernatant solution,

followed by storage at room temperature, yielded a second crop of crystals. Combined yield: 71 mg (20%). Attempts to analyse **2** by NMR spectroscopy in a range of compatible solvents (benzene-d_6, toluene-d_8, tetrahydrofuran-d_8, CD_3CN) were unsuccessful due its highly insoluble nature (at both room temperature and elevated temperatures). **Elemental microanalysis**: calc. for $C_{80}H_{140}N_8Ge_4$ C 63.86%, H 9.38%, N 7.45%, meas. C 63.73%, H 9.54%, N 7.17%.

Synthesis of $(Ar^{NiPr2}Ge)_2Fe(CO)_4$ (3): To a rapidly stirred mixture of $Ar^{NiPr2}GeCl$ (500 mg, 1.21 mmol) and $Na_2Fe(CO)_4$·1.5(1,4-dioxane) (340 mg, 0.98 mmol) at –78 °C was added THF (10 mL). The resulting slurry was stirred at –78 °C for 10 min before being allowed to warm to room temperature and stirred overnight. The resulting dark red solution was filtered and volatiles were removed *in vacuo* to yield a brown oil which was treated with *n*-hexane (100 mL). Filtration of the solution, followed by concentration to the point of incipient crystallisation and storage at –30 °C overnight, yielded single, orange crystals which were suitable for X-ray crystallography. The crystals were isolated and recrystallized from *n*-hexane at –30 °C to remove all minor impurities. Yield: 77 mg (14%). **Spectroscopic data: ^{1}H NMR** (400 MHz, benzene-d_6, 298 K): δ_H 0.98 (12H, d, $^3J_{HH}$ = 6.6 Hz, CH_3 of ^{i}Pr), 1.08 (12H, d, $^3J_{HH}$ = 6.80 Hz, CH_3 of ^{i}Pr), 1.11 – 1.39 (24 H, br. m, CH_3 of ^{i}Pr), 2.80 – 3.73 (12 H, br. m, overlapping C*H* of ^{i}Pr and CH_2N), 3.74 – 4.11 (2 H, br. m, CH_2N), 4.19 (2H, d, $^2J_{HH}$ = 15.3 Hz, CH_2N), 7.27 (1H, t, $^3J_{HH}$ = 7.5 Hz, *p*-C*H* of Ar), 7.32 – 7.76 (1H, br. m, C*H* of Ar). The remaining aromatic C*H* resonances are obscured by the residual solvent resonance. **^{13}C{^{1}H} NMR** (126 MHz, benzene-d_6, 298 K): δ_C 19.8, 21.6 (br.), 23.0 (*C*H_3 of ^{i}Pr), 52.5 (br.) and 55.8 (br.) (*C*H of ^{i}Pr and *C*H_2N), 124.1 (br.), 128.0, 128.4, 129.0, 146.5, 152.1, 157.8 (Ar*C*), 218.4 (*C*O). **IR (nujol/cm^{-1})** υ_{CO}: 2002, 1920, 1897. **Elemental microanalysis**: calc. for $C_{44}H_{70}N_4O_4Ge_2Fe$ C 57.43%, H 7.67%, N 6.09%, meas. C 57.74%, H 7.63%, N 6.16%.

Synthesis of $(Ar^{NiPr2}Ge(IMe_4))_2$ (4): Method A: To a Schlenk tube was added $(Ar^{NiPr2}Ge)_4$ (45 mg, 0.03 mmol), IMe_4 (19 mg, 0.15 mmol) and toluene (2 mL). The resulting slurry was stirred for 18 h at room temperature, during which time the red crystals of $(Ar^{NiPr2}Ge)_4$ dissolved and the solution evolved a bright orange-red colour. Volatiles were subsequently removed *in vacuo* and the resulting red residue was then extracted into minimal diethyl ether, filtered and concentrated to the point of incipient crystallisation. Storage of this solution at −30 °C overnight yielded single, red crystals, which were suitable for X-ray crystallography. Yield: 9 mg (15%). **Method B:** A mixture of $Ar^{NiPr2}GeCl$ (400 mg, 0.97 mmol) and IMe_4 (157 mg, 1.27 mmol) was dissolved in THF (7 mL) and the resulting solution was cooled to –78 °C. To the solution was added dropwise a solution of KNaph (195 mg, 1.17 mmol) in THF (5 mL). Upon completion of the dropwise addition, the orange-red solution was allowed to warm to room temperature and stirred for 2 h. Filtration of the solution followed by removal of volatiles *in vacuo* yielded a viscous red oil which was extracted into minimal diethyl ether, filtered and concentrated to the point of incipient crystallisation. Storage of this solution at –30 °C overnight yielded red crystals, which were isolated and dried *in vacuo*. Further concentration of the supernatant solution, followed by storage at –30 °C, yielded a second crop of crystals. Yield: 114 mg (23%). **^{1}H NMR** (400 MHz, benzene-d_6, 298 K): δ_H 0.88 – 1.32 (48H, br. m, CH_3 of ^{i}Pr), 1.52 (12 H, s, C–CH_3,

NHC), 3.03 – 3.25 (8H, br. m, C*H* of ^{i}Pr), 3.33 (12 H, s, N–CH_3, NHC), 3.68 – 4.90 (8H, br. m, CH_2N), 7.43 (2H, t, $^3J_{HH}$ = 7.5 Hz, *p*-C*H* of Ar), 7.77 – 8.18 (4H, br. m, *m*-C*H* of Ar). **^{13}C{^{1}H} NMR** (101 MHz, benzene-d$_6$, 298 K): δ_C 8.6 ($C^{4,5}$-CH_3, NHC), 21.5 (CH_3 of ^{i}Pr), 34.5 (N–CH_3, NHC), 47.8 (*C*H of ^{i}Pr), 124.3 (overlapping resonances for *m*-Ar*C* and $C_{4,5}$–CH_3 of NHC), 124.7 (*p*-Ar*C*), 148.8 (*o*-Ar*C*), 158.7 (*ipso*-Ar*C*), 180.5 (Ge-*C*, NHC). The resonances for *C*H$_2$N of Ar^{NiPr2} are not observed. **Elemental microanalysis**: calc. for $C_{54}H_{94}N_8Ge_2$ C 64.82%, H 9.47%, N 11.20%, meas. C 64.48%, H 9.51%, N 10.96%.

Synthesis of (Ar^{NiPr2}Ge{W(CO)$_5$})$_2$ (5): To a mixture of Ar^{NiPr2}GeCl (200 mg, 0.49 mmol) and (^{Mes}NacnacMg)$_2$ (209 mg, 0.29 mmol) was added benzene (7 mL). The resulting slurry was stirred for 1 h at room temperature and was subsequently filtered into a Schlenk tube containing W(CO)$_5$(NMe$_3$) (223 mg, 0.58 mmol). The solution was then heated to 75 °C for 20 h* before volatiles were removed *in vacuo* to yield an oily orange residue. The residue was washed with *n*-hexane (5 mL) and then extracted with toluene (10 mL). The resulting solution was then filtered and concentrated to the point of incipient crystallisation. Storage of the solution at –30 °C for 3 d yielded yellow crystals of **5** which were isolated and dried *in vacuo*. Yield: 102 mg (30%). Crystals of **5** (as the benzene solvate) which were suitable for X-ray crystallography were obtained from storage of a concentrated solution of **5** in benzene at room temperature. **Spectroscopic data: ^{1}H NMR** (400 MHz, benzene-d$_6$, 298 K): δ_H 0.33 (3H, d, $^3J_{HH}$ = 6.8 Hz, CH_3 of ^{i}Pr), 0.65 (3H, d, $^3J_{HH}$ = 6.3 Hz, CH_3 of ^{i}Pr), 0.90 (6H, d, $^3J_{HH}$ = 6.5 Hz, CH_3 of ^{i}Pr), 0.94 (3H, d, $^3J_{HH}$ = 6.8 Hz, CH_3 of ^{i}Pr), 1.00 (6H, d, $^3J_{HH}$ = 6.7 Hz, CH_3 of ^{i}Pr), 1.06 (6H, d, $^3J_{HH}$ = 6.7 Hz, CH_3 of ^{i}Pr), 1.34 (3H, d, $^3J_{HH}$ = 7.1 Hz, CH_3 of ^{i}Pr), 2.88 – 2.92 (3H, m, overlapping resonances for C*H* of ^{i}Pr and CH_2N), 3.00 and 3.14 (2H, AB system, $^2J_{HH}$ = 14.6 Hz, CH_2N), 3.26 – 3.34 (4H, m, overlapping resonances for C*H* of ^{i}Pr and CH_2N), 3.61 and 3.82 (2H, AB system, $^2J_{HH}$ = 14.6 Hz, CH_2N), 3.75 (1H, sept, $^3J_{HH}$ = 6.7 Hz, C*H* of ^{i}Pr), 4.30 – 4.40 (2H, m, CH_2N), 6.70 (1H, d, $^3J_{HH}$ = 7.6 Hz, *m*-C*H* of Ar), 6.75 (1H, d, $^3J_{HH}$ = 7.6 Hz, *m*-C*H* of Ar), 7.04 (1H, t, $^3J_{HH}$ = 7.5 Hz, *p*-C*H* of Ar), 7.26 (1H, t, $^3J_{HH}$ = 7.5 Hz, *p*-C*H* of Ar), 7.56 (1H, d, $^3J_{HH}$ = 7.6 Hz, *m*-C*H* of Ar), 8.03 (1H, d, $^3J_{HH}$ = 7.7 Hz, *m*-C*H* of Ar). Resonance(s) for 2 x CH_3 of ^{i}Pr (6H) are not observed in the ^{1}H NMR spectrum. **^{13}C{^{1}H} NMR** (126 MHz, benzene-d$_6$, 298 K): δ_C 18.4 (CH_3 of ^{i}Pr), 19.6 (2 x CH_3 of ^{i}Pr), 20.0, 21.5, 22.6, 22.9, 24.1 (CH_3 of ^{i}Pr), 48.1 (br.), 48.6 (br.) (*C*H of ^{i}Pr), 50.6 (br.) (CH_2N), 52.0 (CH_2N), 54.7 (*C*H of ^{i}Pr), 56.3 (CH_2N), 59.0 (*C*H of ^{i}Pr), 61.6 (br.) (CH_2N), 120.5, 121.1 (br.), 126.0, 127.0, 127.5 (br.), 129.2, 144.7, 145.4, 147.3, 148.9, 149.0, 162.0 (Ar*C*), 203.4, 203.6 (*C*O). **IR (nujol/cm^{-1})** υ_{CO}: 2043, 1918, 1908, 1896.

*These reaction conditions were established by monitoring the reaction of crystalline (Ar^{NiPr2}Ge)$_4$ (10 mg, 0.007 mmol) and W(CO)$_5$(NMe$_3$) (10 mg, 0.026 mmol) in benzene-d$_6$ (0.5 mL) by ^{1}H NMR spectroscopy.

Synthesis of (Ar^{NEt2}GeGeAr^{NiPr2})$_2$ (6): To an NMR tube fitted with a J. Young's valve was added Ar^{NEt2}GeCl (13 mg, 0.037 mmol), Ar^{NiPr2}GeCl (15 mg, 0.036 mmol) and benzene-d$_6$ (0.5 mL). After this, [(^{Mes}Nacnac)Mg]$_2$ (27 mg, 0.038 mmol) was added and the resulting reaction mixture was shaken vigorously, leading to a change in colour of the solution (from pale yellow to deep red) and the

precipitation of a colourless powder. Storage of the reaction mixture at room temperature for 48 h led to the formation of a small number of single, dark red crystals which were suitable for X-ray crystallography.

Reaction of (Ar^{NiPr2}Ge{IMe_4})$_2$ (4) and BPh_3: To a solution of (Ar^{NiPr2}Ge(IMe_4))$_2$ (0.02 g, 0.020 mmol) in benzene-d_6 (0.5 mL) in an NMR tube fitted with a J. Young's valve was added BPh_3 (0.01 g, 0.041 mmol). Upon addition of the Lewis acid, the orange-red solution immediately changed colour to deep red. The resulting solution was analysed by ^{1}H and ^{11}B NMR spectroscopy which showed that complete consumption of (Ar^{NiPr2}Ge(IMe_4))$_2$ occurs, accompanied by formation of $IMe_4 \cdot BPh_3$; ^{1}H and ^{11}B NMR data are consistent with that reported by Inoue *et al.*[S8] Over the course of 12 h, dark red powder precipitated from the benzene solution. Dark red crystals of (Ar^{NiPr2}Ge)$_4$ could be grown from the solution by redissolving this powder at 60 °C and allowing the solution to cool slowly to room temperature; the identity of the crystals was confirmed by analysis by X-ray crystallography.

(iv) ^{1}H and ^{13}C NMR spectra of novel compounds

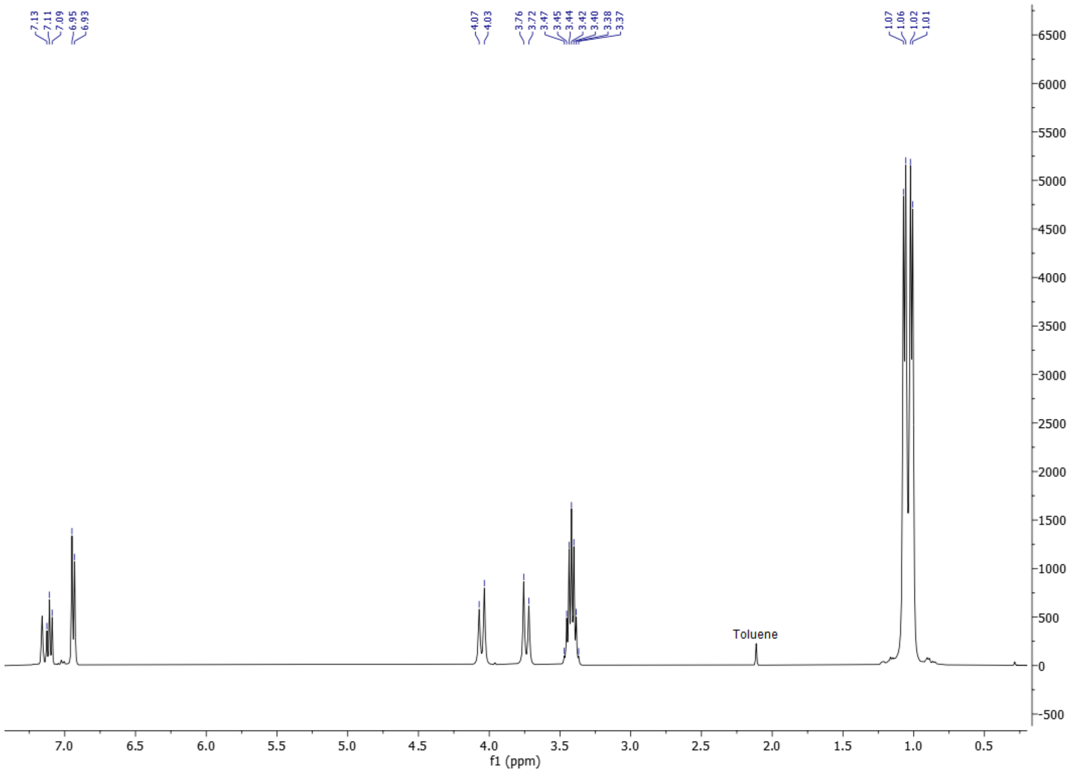

Figure S1. ^{1}H NMR spectrum of $Ar^{NiPr2}GeI$ measured in benzene-d_6 at 298 K. Toluene impurity has been labelled.

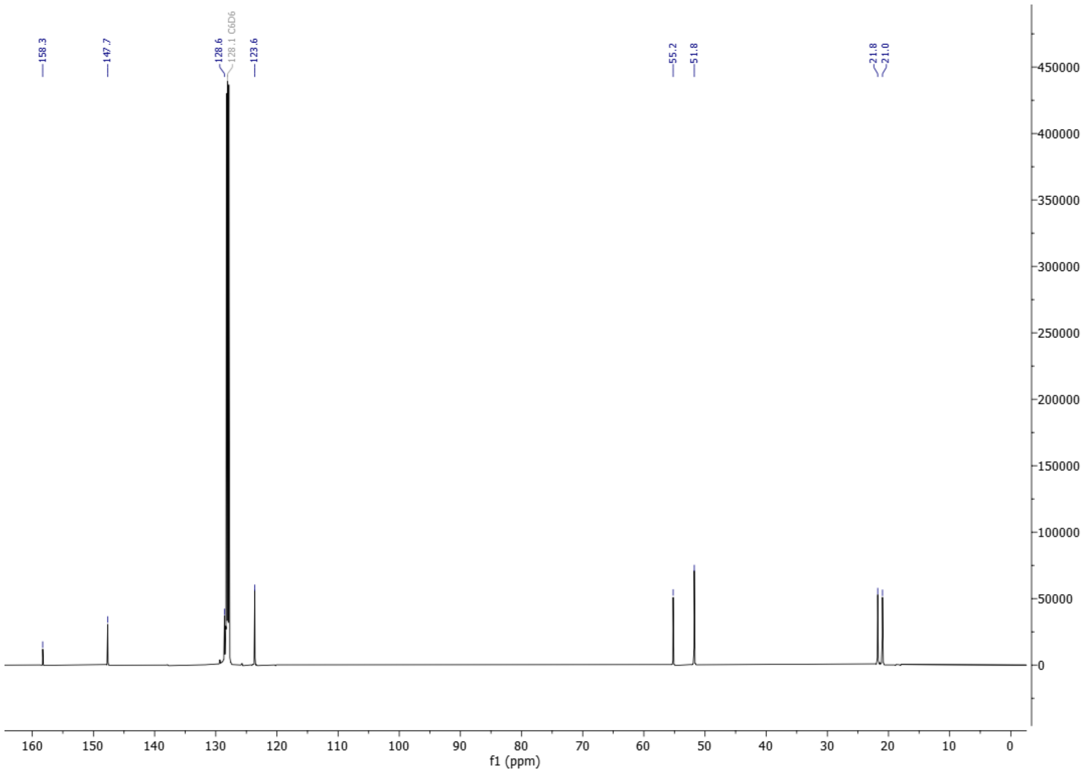

Figure S2. ^{13}C NMR spectrum of $Ar^{NiPr2}GeI$ measured in benzene-d_6 at 298 K.

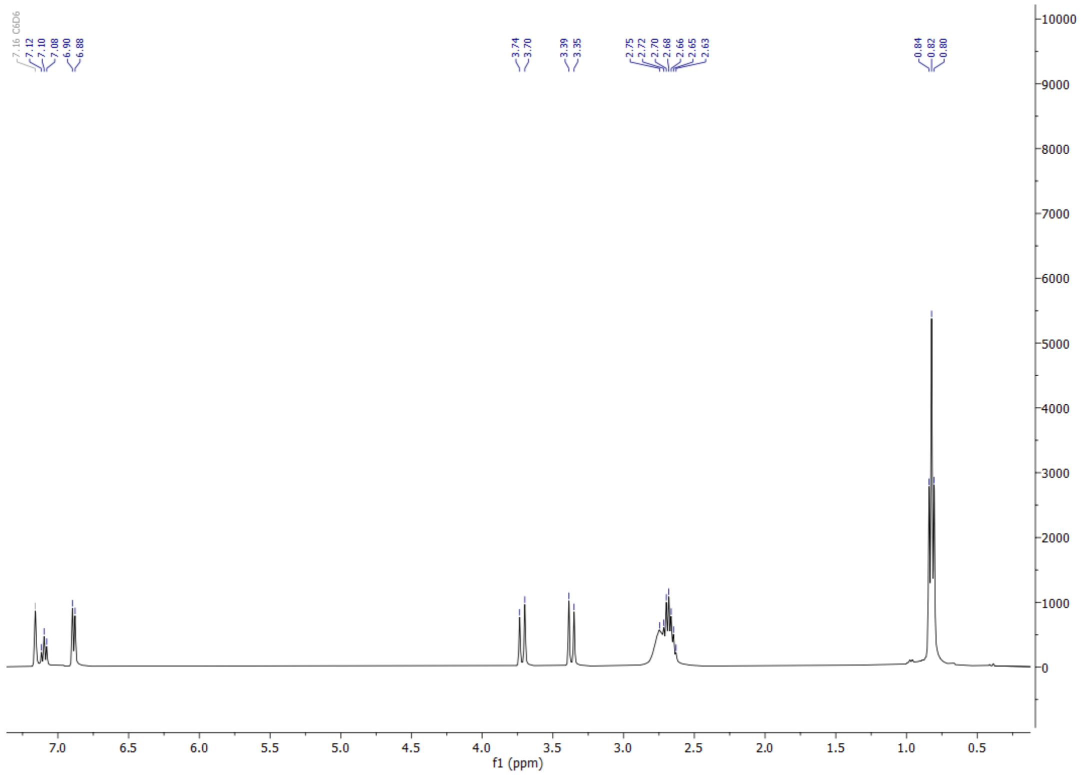

Figure S3. ^{1}H NMR spectrum of $Ar^{NEt2}GeI$ measured in benzene-d_6 at 298 K.

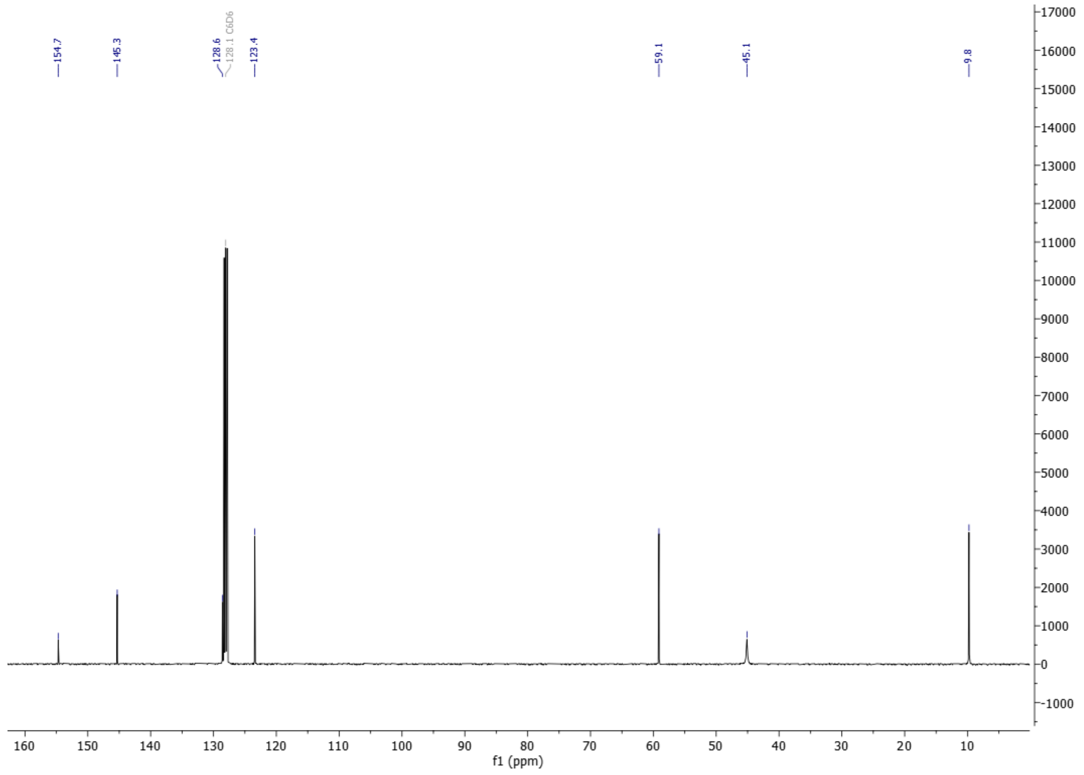

Figure S4. ^{13}C NMR spectrum of $Ar^{NEt2}GeI$ measured in benzene-d_6 at 298 K.

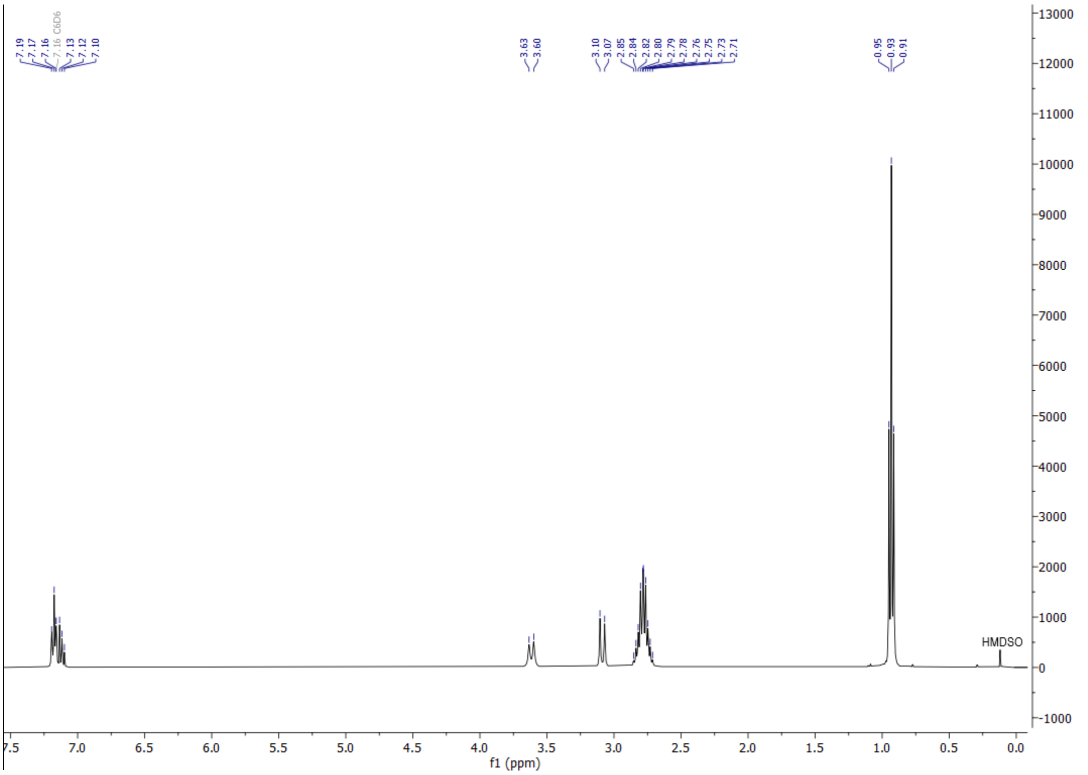

Figure S5. ^{1}H NMR spectrum of $(Ar^{NEt2}Ge)_2$ (**1**) measured in benzene-d_6 at 298 K. HMDSO impurity has been labelled.

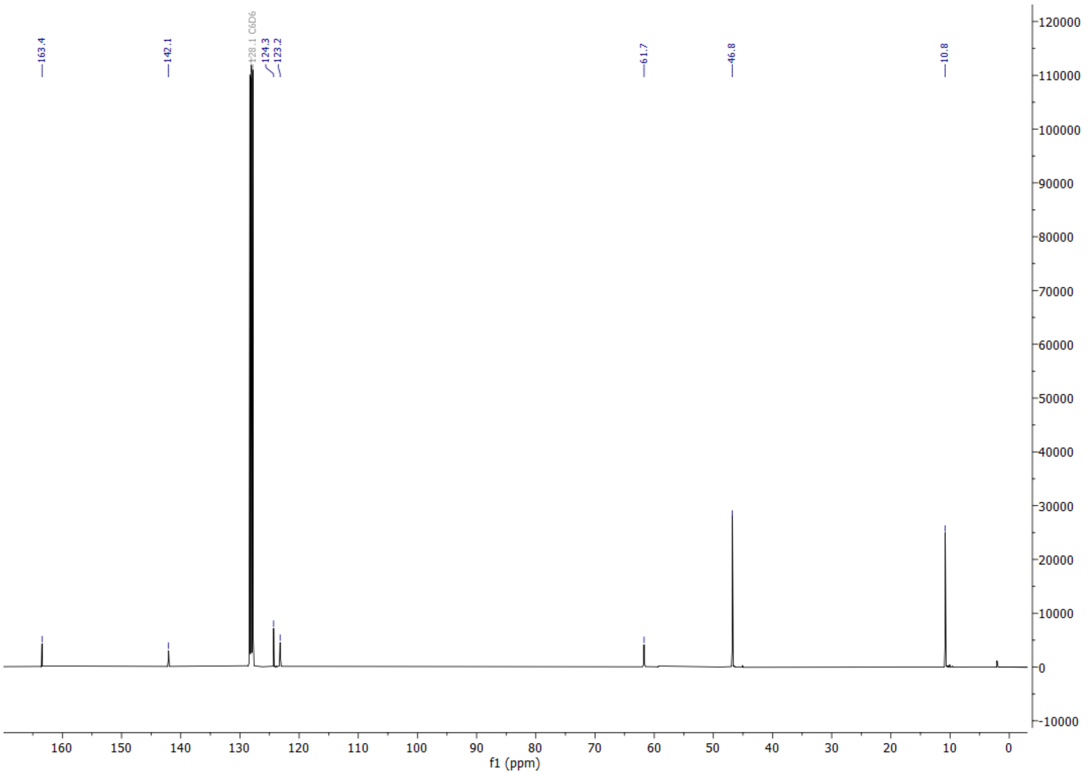

Figure S6. ^{13}C NMR spectrum of $(Ar^{NEt2}Ge)_2$ (**1**) measured in benzene-d_6 at 298 K.

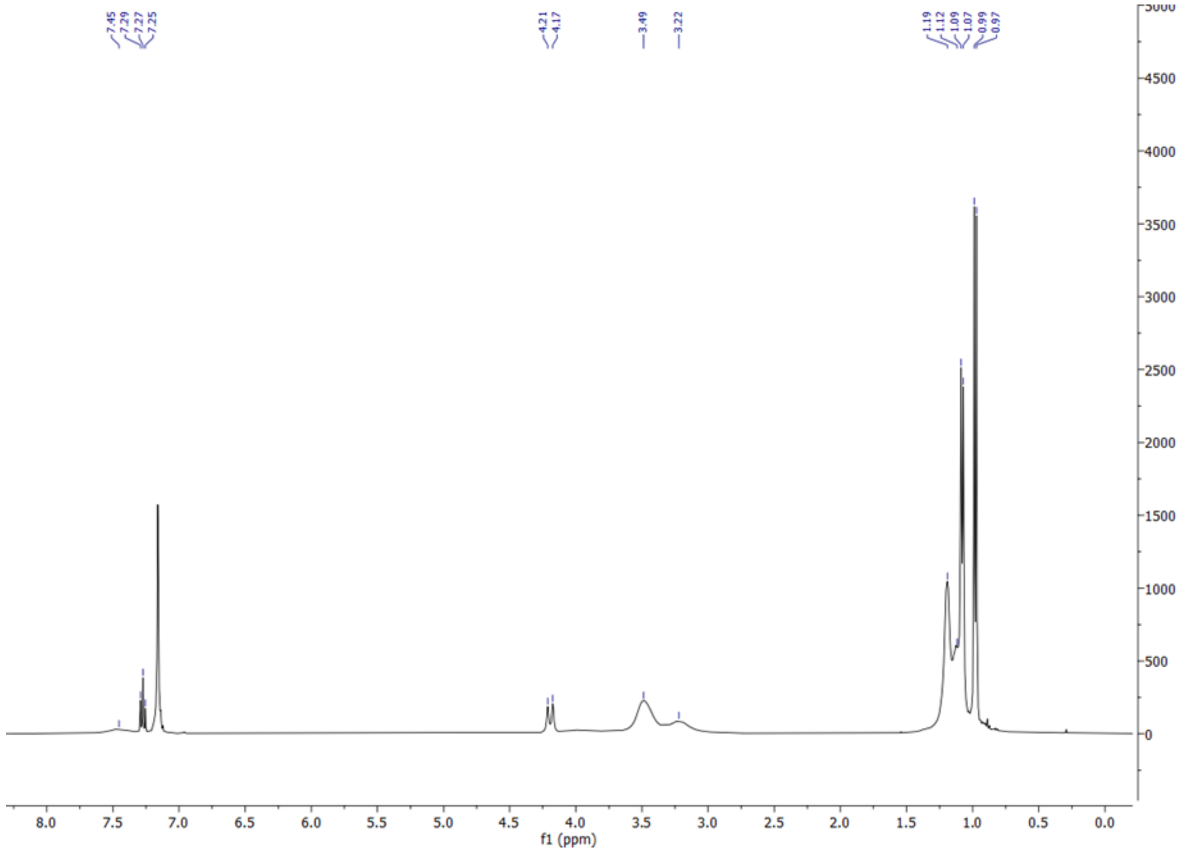

Figure S7. ^{1}H NMR spectrum of $(Ar^{NiPr2}Ge)_2Fe(CO)_4$ (**3**) measured in benzene-d$_6$ at 298 K.

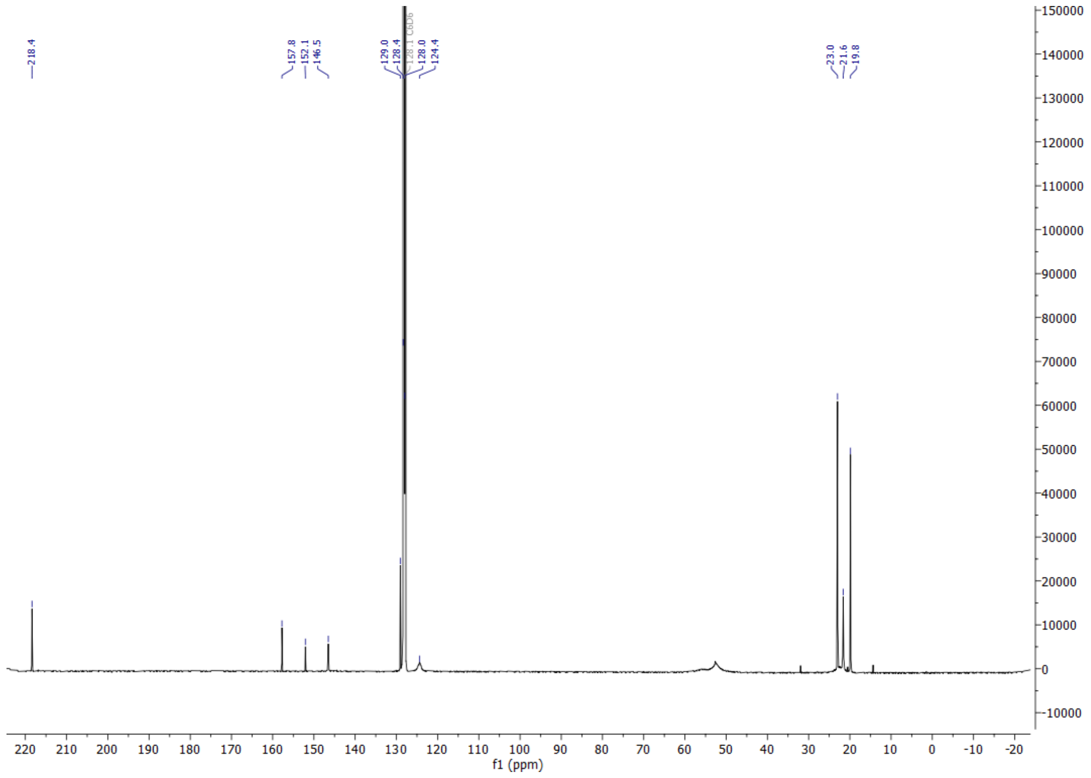

Figure S8. ^{13}C NMR spectrum of $(Ar^{NiPr2}Ge)_2Fe(CO)_4$ (**3**) measured in benzene-d$_6$ at 298 K.

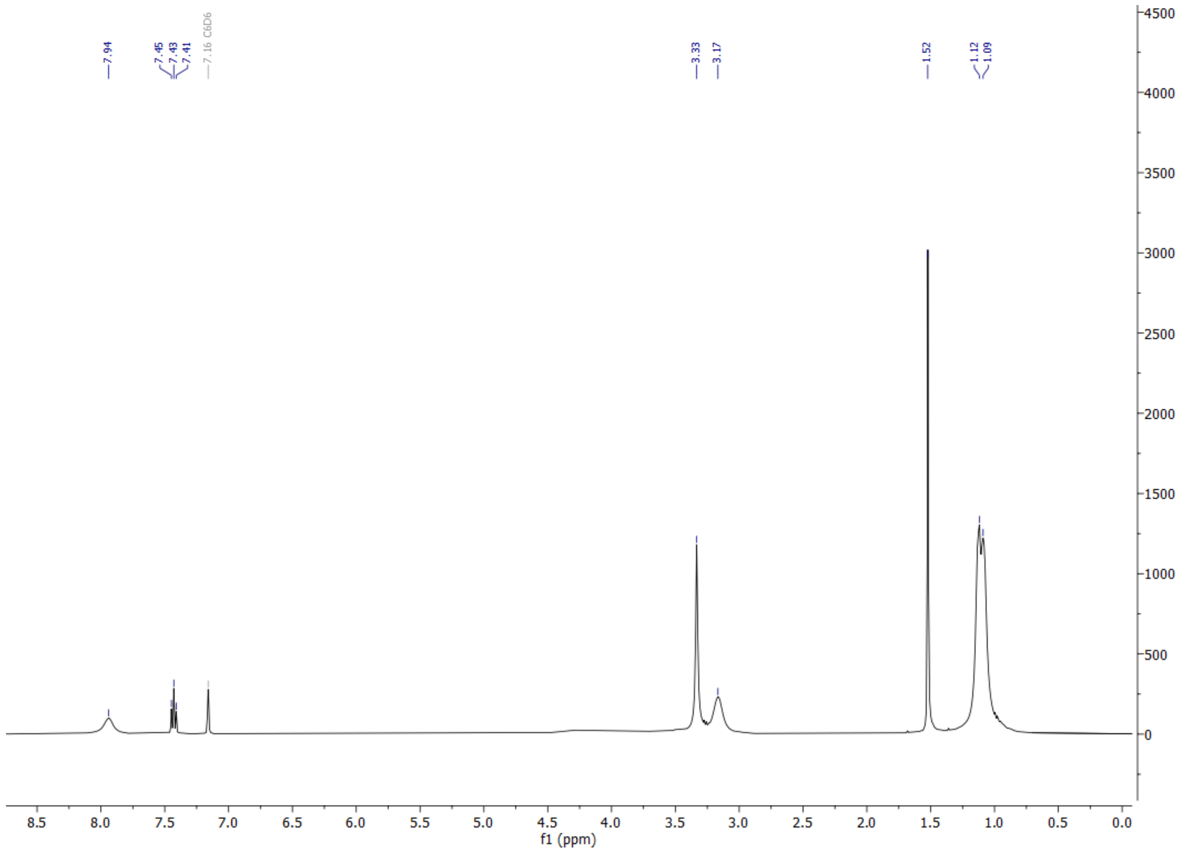

Figure S9. ^{1}H NMR spectrum of ((Ar^{NiPr2}Ge(IMe$_4$))$_2$ (**4**) measured in benzene-d$_6$ at 298 K.

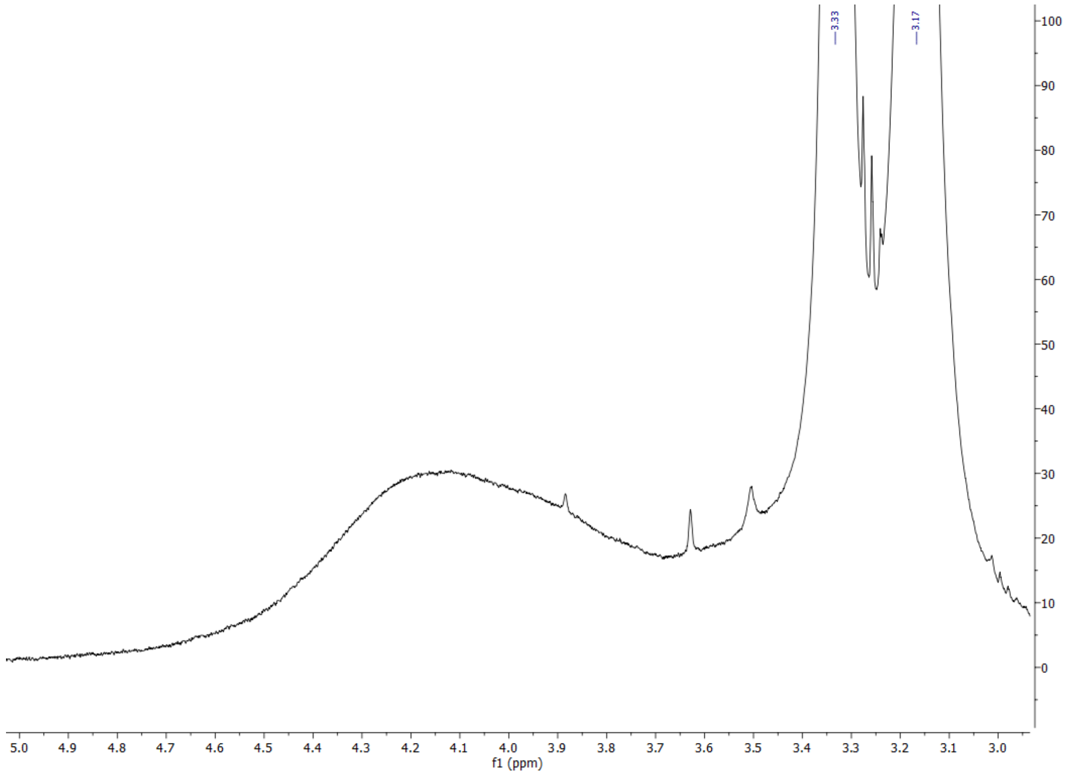

Figure S10. ^{1}H NMR spectrum of ((Ar^{NiPr2}Ge(IMe$_4$))$_2$ (**4**) measured in benzene-d$_6$ at 298 K in region *ca.* 3.0 – 5.0 ppm.

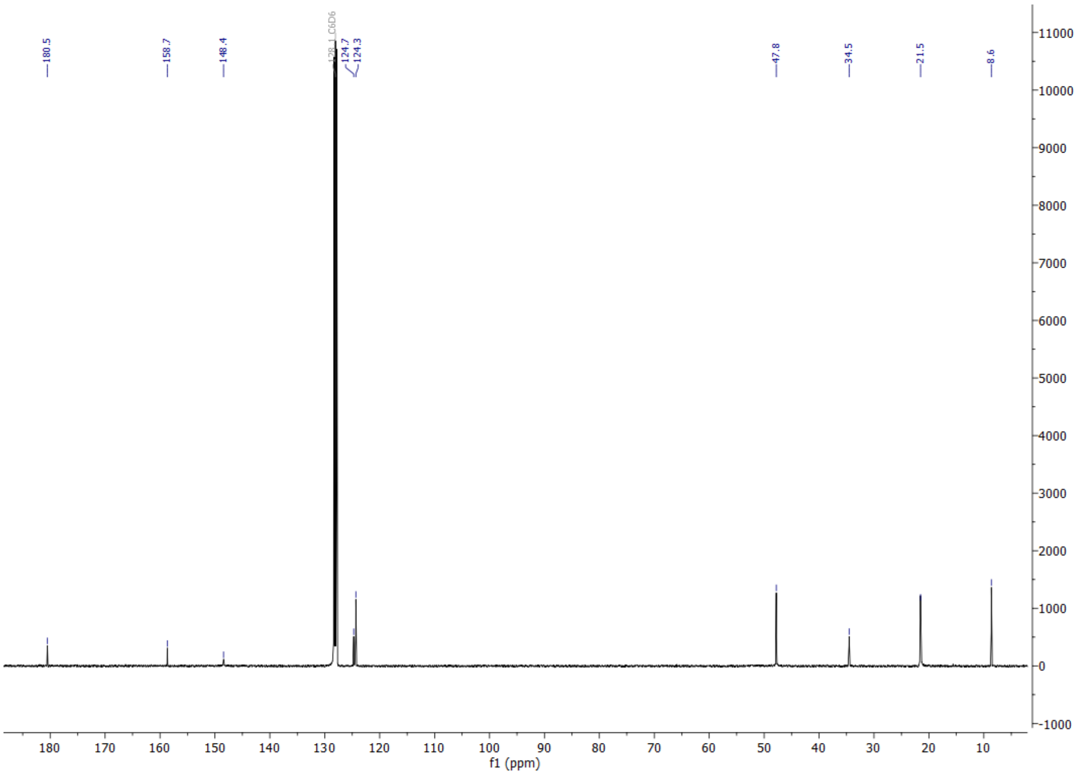

Figure S11. ^{13}C NMR spectrum of $((Ar^{NiPr2}Ge(IMe_4))_2$ (**4**) measured in benzene-d_6 at 298 K.

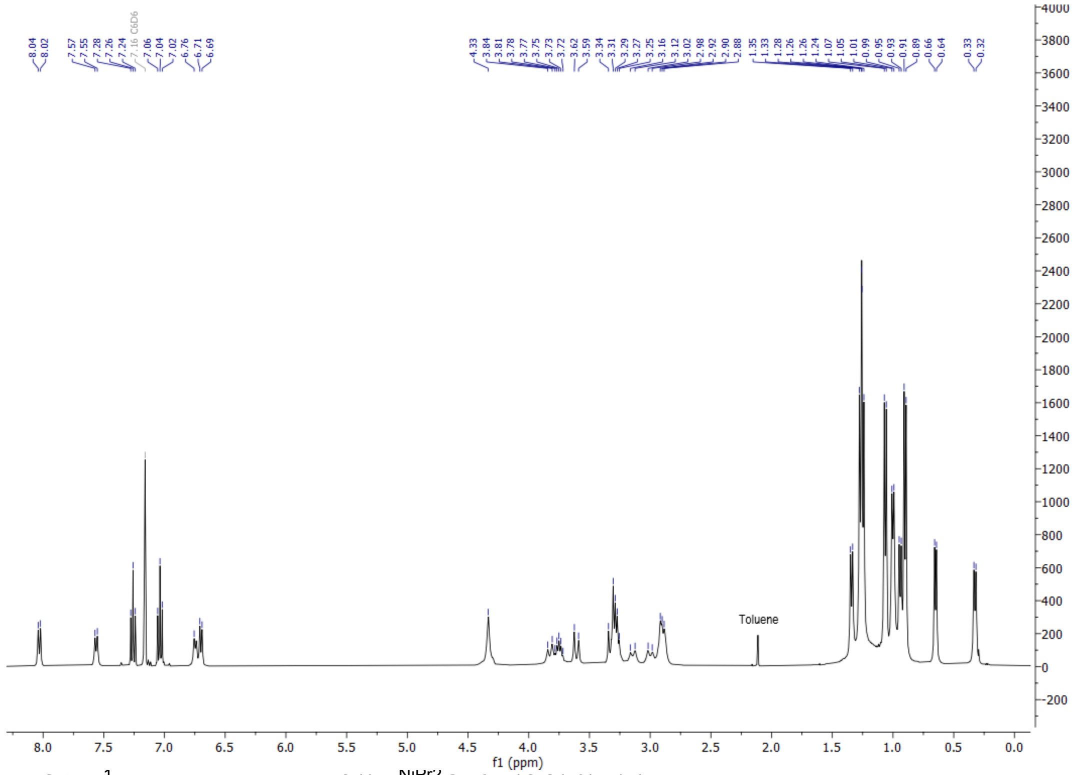

Figure S12. ^{1}H NMR spectrum of $((Ar^{NiPr2}Ge\{W(CO)_5\})_2$ (**5**) measured in benzene-d$_6$ at 298 K. Toluene impurity has been labelled.

(v) X-ray crystallographic studies

Single-crystal X-ray diffraction data for all compounds were collected at 150 K on an Oxford Diffraction/Agilent SuperNova diffractometer using Cu-K_{α} radiation (λ = 1.54184 Å) or Mo-K_{α} (λ = 0.71073 Å), and equipped with a nitrogen gas Oxford Cryosystems cooling unit.[S9] Raw frame data were reduced using CrysAlisPro.[S10] The structures were solved using SHELXT[S11] and refined to convergence on F^2 by full-matrix least-squares using SHELXL[S12] in combination with OLEX2.[S13] Distances and angles were calculated using the full covariance matrix. Restraints were used to maintain sensible geometries for the disordered groups and approximate the displacement parameters to typical values. Selected crystallographic data are summarized in Table S2 and full details are given in the supplementary deposited CIF files (CCDC 2075218-2075226). These data can be obtained free of charge from the Cambridge Crystallographic Data Centre via http://www.ccdc.cam.ac.uk/data_request/cif.

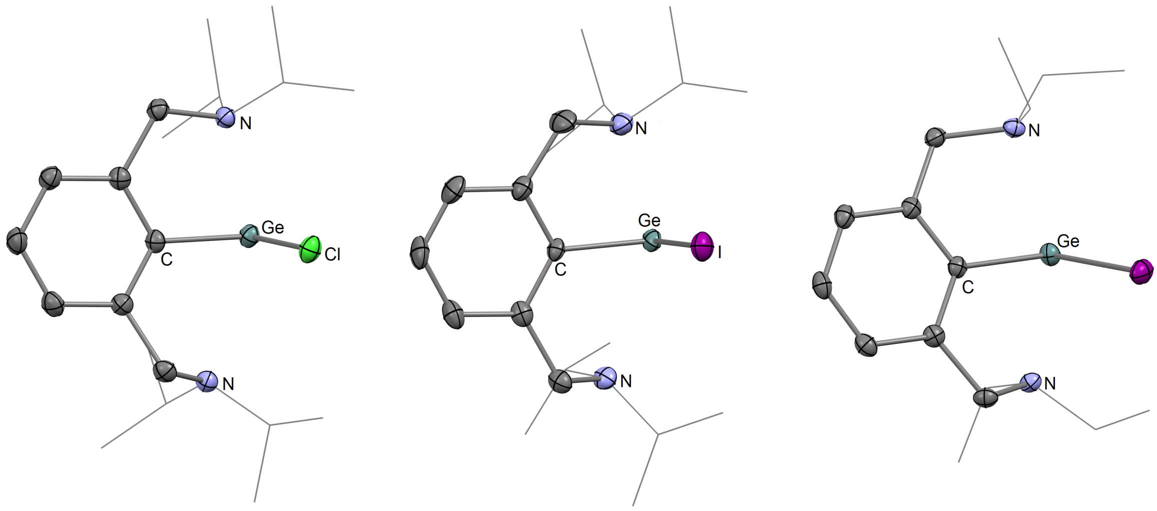

Figure S14. The molecular structures of Ar^{NiPr2}GeCl (left), Ar^{NiPr2}GeI (middle) and Ar^{NEt2}GeI (right). Thermal ellipsoids set at the 40% probability level. Hydrogen atoms omitted and ^{i}Pr/Et substituents shown in wireframe format for clarity.

Table S1. Selected bond lengths and angles for Ar^{NiPr2}GeCl, Ar^{NiPr2}GeI and Ar^{NEt2}GeI.

	Ar^{NiPr2}GeCl	Ar^{NiPr2}GeI	Ar^{NEt2}GeI
d(Ge–Hal) (Å)	2.3162(7)	2.7448(6)	2.7052(4)
d(Ge–C) (Å)	1.990(2)	1.983(3)	1.977(2)
d(Ge–N) (Å)	2.315(2)/2.892(2)	2.310(3)/2.851(3)	2.393(2)/2.530(2)
C–Ge–Hal (°)	94.33(7)	89.36(9)	98.62(6)

Table S2. Selected X-ray data collection and refinement parameters

	$Ar^{NiPr2}GeCl$	$Ar^{NiPr2}GeI$	$Ar^{NEt2}GeI$
Formula	$C_{20}H_{35}Cl_1Ge_1N_2$	$C_{20}H_{35}GeIN_2$	$C_{16}H_{27}GeIN_2$
Fw (g mol^{-1})	411.55	502.99	446.88
Cell setting	Monoclinic	Orthorhombic	Orthorhombic
Space group	$P2_1/n$	*P b c a*	*P b c a*
a (Å)	16.1396(3)	10.1974(2)	9.4583(2)
b (Å)	7.6686(2)	16.0545(2)	13.5229(2)
c (Å)	16.8862(3)	26.8797(4)	28.2593(5)
α (°)	90	90	90
β (°)	90.6090(10)	90	90
γ (°)	90	90	90
V (Å^3)	2089.86(8)	4400.59(12)	3614.47(11)
Z	4	8	8
ρ_{calc} (g cm^{-3})	1.308	1.518	1.642
Radiation, λ (Å)	Mo K_α 0.71073	Cu K_α 1.54184	Mo K_α 0.71073
μ (mm^{-1})	1.598	12.918	3.397
Reflections collected	41492	14786	30956
Independent reflections	4738	4537	4863
$R_{(int)}$	0.033	0.0369	0.0485
Parameters	218	255	184
R_1 (all data/$I > 2\sigma(I)$)	0.0515/0.0356	0.0485/0.0404	0.0443/0.0281
ωR_2 (all data/$I > 2\sigma(I)$)	0.0909/0.0762	0.1137/0.1058	0.0613/0.0551
GooF	0.9346	1.019	1.043
T/K	150(2)	150(2)	150.00(10)
CCDC Deposition No.	2075220	2075226	2075224

Table S2 (cont). Selected X-ray data collection and refinement parameters for compounds **1 - 3**

	$(Ar^{NEt2}Ge)_2$ (**1**)	$(Ar^{NiPr2}Ge)_4$ (**2**)	$(Ar^{NiPr2}Ge)_2Fe(CO)_4{\cdot}C_6H_{14}$ (**3**)
Formula	$C_{32}H_{54}Ge_2N_4$	$C_{80}H_{140}Ge_4N_8$	$C_{44}H_{70}FeGe_2N_4O_4{\cdot}C_6H_{14}$
Fw (g mol^{-1})	639.97	1504.35	1006.24
Cell setting	Triclinic	Triclinic	Triclinic
Space group	*P*-1	*P*-1	*P*-1
a (Å)	10.0623(2)	12.7216(4)	10.9188(2)
b (Å)	10.2095(3)	12.9110(5)	12.4696(3)
c (Å)	17.5174(5)	13.6719(6)	20.2958(5)
α (°)	98.262(3)	110.994(4)	82.201(2)
β (°)	96.218(2)	99.280(3)	75.014(2)
γ (°)	110.242(2)	93.034(3)	84.194(2)
V (Å^3)	1646.53(8)	2054.10(15)	2638.35(11)
Z	2	1	2
ρ_{calc} (g cm^{-3})	1.291	1.216	1.267
Radiation, λ (Å)	Cu K_α 1.54184	Cu K_α 1.54184	Mo K_α 0.71073
μ (mm^{-1})	2.427	2.017	1.447
Reflections collected	26435	16142	54764
Independent reflections	6806	7054	14475
$R_{(int)}$	0.0562	0.0201	0.0401
Parameters	351	431	644
R_1 (all data/$I > 2\sigma(I)$)	0.0446/0.0346	0.0322/0.0293	0.0782/0.0518
ωR_2 (all data/$I > 2\sigma(I)$)	0.0918/0.0845	0.0758/0.0735	0.1410/0.1251
GooF	1.031	1.020	1.030
T/K	150.01(11)	150(2)	150.01(16)
CCDC Deposition No.	2075218	2075219	2075223

Table S2 (cont). Selected X-ray data collection and refinement parameters for compounds **4 - 6**

	$(Ar^{NiPr2}Ge(IMe_4))_2$ (**4**)	$(Ar^{NiPr2}Ge\{W(CO)_5\})_2 \cdot C_6H_6$ (**5**)	$(Ar^{NEt2}GeGeAr^{NiPr2})_2$ (**6**)
Formula	$C_{54}H_{94}Ge_2N_8$	$C_{50}H_{70}Ge_2N_4O_{10}W_2 \cdot C_6H_6$	$C_{36}H_{61}Ge_2N_4$
Fw (g mol^{-1})	1000.55	1478.08	695.14
Cell setting	Monoclinic	Triclinic	Triclinic
Space group	*P* 1 21/*c* 1	*P*-1	*P*-1
a (Å)	14.1000(3)	12.5281(2)	12.5765(10)
b (Å)	26.6880(8)	12.5540(3)	13.0464(11)
c (Å)	16.2808(4)	19.5249(4)	13.4226(11)
α (°)	90	103.019(2)	115.125(8)
β (°)	109.404(3)	91.692(2)	100.907(7)
γ (°)	90	100.321(2)	92.679(7)
V (Å^3)	5778.5(3)	2935.65(11)	1937.8(3)
Z	4	2	2
ρ_{calc} (g cm^{-3})	1.150	1.672	1.1912
Radiation, λ (Å)	Cu K_α 1.54184	Cu K_α 1.54184	Cu K_α 1.54184
μ (mm^{-1})	1.570	8.706	2.101
Reflections collected	33132	55627	15811
Independent reflections	11906	12280	7997
$R_{(int)}$	0.0969	0.0264	0.0406
Parameters	601	683	422
R_1 (all data/$I > 2\sigma(I)$)	0.0896/0.0604	0.0229/0.0181	0.0897/0.0619
ωR_2 (all data/$I > 2\sigma(I)$)	0.1732/0.1471	0.0419/0.0399	0.1907/0.1600
GooF	0.994	1.048	1.0400
T/K	150.01(18)	150.01(10)	150.01(10)
CCDC Deposition No.	2075225	2075221	2075222

(vi) References

[S1] G. A. Carriedo, J. A. K. Howard, J. C. Jeffery, K. Sneller, F. G. A. Stone, A. M. M. Weerasuria, *J. Chem. Soc. Dalt. Trans.* **1990**, 953–958.

[S2] C. Bibal, S. Mazières, H. Gornitzka, C. Couret, *Polyhedron* **2002**, *21*, 2827–2834.

[S3] S. J. Bonyhady, C. Jones, S. Nembenna, A. Stasch, A. J. Edwards, G. J. McIntyre, *Chem. - A Eur. J.* **2010**, *16*, 938–955.

[S4] B. A. Merrill, in *Encycl. Reagents Org. Synth.*, John Wiley & Sons, Ltd, Chichester, UK, **2001**.

[S5] A. Weitz, M. Rabinovitz, *Synth. Met.* **1995**, *74*, 201–205.

[S6] S. L. Matthews, D. M. Heinekey, *J. Am. Chem. Soc.* **2006**, *128*, 2615–2620.

[S7] N. Kuhn, T. Kratz, *Synthesis* **1993**, *6*, 561–562

[S8] D. Sarkar, V. Nesterov, T. Szilvási, P. J. Altmann, S. Inoue, *Chem. - A Eur. J.* **2019**, *25*, 1198–1202.

[S9] J. Cosier, A. M. Glazer, *J. Appl. Cryst.* **1986**, *19*, 105-107.

[S10] CrysAlisPRO, Oxford Diffraction/Agilent Technologies UK Ltd, Yarnton, UK

[S11] G. Sheldrick, *Acta Cryst. C* **2015**, *71*, 3-8.

[S12] G. Sheldrick, *Acta Cryst. A* **2008**, *64*, 112-122.

[S13] O. V. Dolomanov, L. J. Bourhis, R. J. Gildea, J. A. K. Howard, H. Puschmann. *J. Appl. Cryst.* **2009**, *42*, 339-341.
